# Chemical and Enzymatic Methods for Post-Translational Protein–Protein Conjugation

**DOI:** 10.1021/jacs.2c00129

**Published:** 2022-08-01

**Authors:** Ross J. Taylor, Michael B. Geeson, Toby Journeaux, Gonçalo J. L. Bernardes

**Affiliations:** † Department of Chemistry, University of Cambridge, Lensfield Road, CB2 1EW Cambridge, U.K.; ‡ Instituto de Medicina Molecular João Lobo Antunes, Faculdade de Medicina, Universidade de Lisboa, Avenida Professor Egas Moniz, 1649-028, Lisboa, Portugal

## Abstract

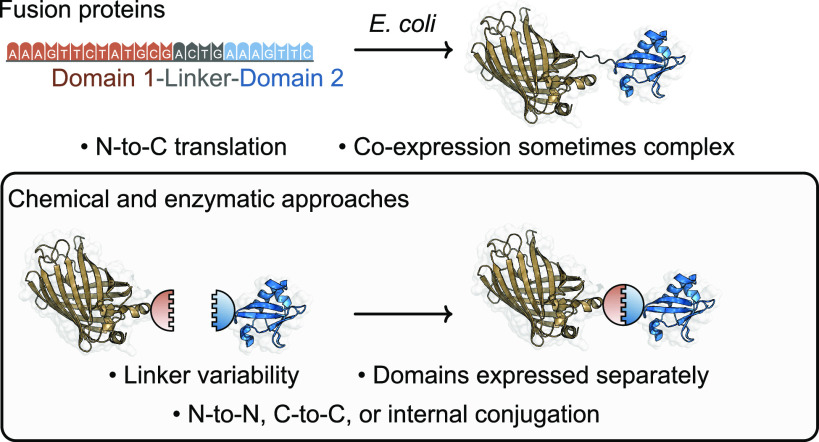

Fusion proteins play an essential role in the biosciences but suffer from several key limitations, including the requirement for N-to-C terminal ligation, incompatibility of constituent domains, incorrect folding, and loss of biological activity. This perspective focuses on chemical and enzymatic approaches for the post-translational generation of well-defined protein–protein conjugates, which overcome some of the limitations faced by traditional fusion techniques. Methods discussed range from chemical modification of nucleophilic canonical amino acid residues to incorporation of unnatural amino acid residues and a range of enzymatic methods, including sortase-mediated ligation. Through summarizing the progress in this rapidly growing field, the key successes and challenges associated with using chemical and enzymatic approaches are highlighted and areas requiring further development are discussed.

## 1 Introduction

**Figure 1:**
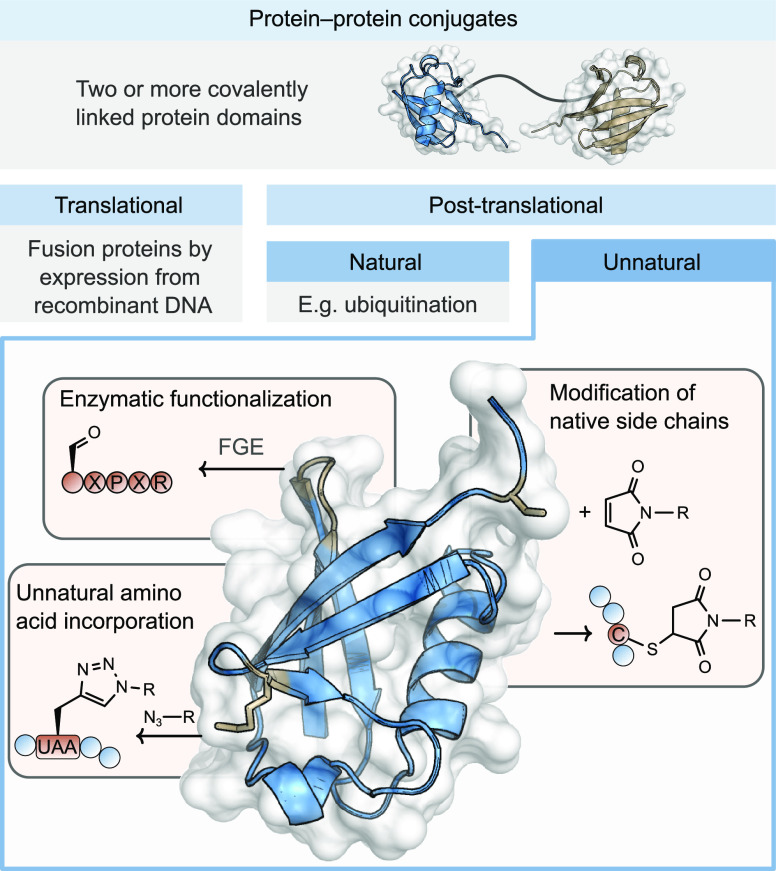
Overview of methods used to prepare unnatural protein–protein conjugates in the context of all available pathways.

Protein–protein conjugates are biomolecules generated from two or more protein domains. The ability to place proteins with mutually exclusive functions in the same location at the same time has the potential to yield properties that would otherwise be impossible to achieve when compared to using component protein monomers in isolation. These biomolecules have a diverse range of applications in the fields of biotechnology and biopharmaceutical research. Nature has evolved numerous post-translational protein–protein conjugates with a key example being the covalent conjugation of multiple ubiquitin subunits to protein substrates, tagging them for degradation by the ubiquitin–proteasome pathway, and in turn regulating cellular processes or clearing aberrant proteins.^1^ Post-translational ubiquitination has been particularly well-studied (Figure 1), and therefore synthetic methods for ubiquitination and subsequent applications are not discussed further herein.^2−4^

An indispensable method for generating protein–protein conjugates has been via so-called “fusion proteins”, generated by translation of a designed DNA sequence. The products of this technology have found applications in protein purification, imaging, and in the production of bifunctional engineered enzymes and bispecific antibodies.^5−9^ Genetic fusion proteins are produced by expression of a gene and result in a single polypeptide chain. The “linker” is the portion of the polypeptide chain that resides between the two protein domains, and its physical characteristics are determined by its constituent amino acids; properties such as flexibility, length, or ability to be cleaved in vivo can be tuned by changing the linker.^10^ It is clear that genetic fusion is a powerful technique for generating protein–protein conjugates, as evidenced by their diverse range of applications. Nonetheless, there are some key limitations faced when using these approaches, which have the potential to be overcome using alternative, post-translational, or “synthetic” conjugation methods. Limitations of recombinantly expressed fusion proteins can include poor yields, incorrect folding, poor stability, and the restrictive necessity of N-to-C terminal fusion, which is particularly challenging in cases when free termini are required to retain biological activity, an area where chemical and enzymatic processes can play a key role.^5,11,12^

In addition, recombinant expression of certain fusion proteins is not feasible, as they may require separate cell lines for expression, as is the case with some immunotoxin conjugates.^13^ By obviating the need for genetic fusion, each protein domain can be expressed independently and subsequently ligated using chemical or biochemical conjugation strategies (Figure 1). In line with the rapidly expanding toolbox for site-selective (targeting a single type of amino acid residue) and site-specific (targeting a single amino acid residue over all others in the protein) protein modification, methods for synthetically generating protein–protein conjugates have seen significant advancements.^14−17^

## 2 The Protein–Protein Coupling Problem

The biggest challenge facing the preparation of protein–protein conjugates is one of kinetics. In traditional bioconjugation reactions between a protein and a small molecule, a common strategy is to use a high stoichiometric excess of the latter in order to increase reaction velocity. This is necessary because proteins are generally present in low concentrations (1–100 μM) and are also large in size, rendering them sterically encumbered coupling partners.^18,19^ However, in the case of protein–protein coupling reactions, it is generally not practical to use a larger stoichiometric excess of one partner. The protein–protein coupling problem arises because two of these sterically encumbered coupling partners, both present at low concentrations, must come together to form the desired protein–protein conjugate.

Naturally, this problem has been addressed by using reactions that have high second-order rate constants (*k*_2_).^20^ Therefore, a common theme is the inclusion of functional groups for “click chemistry”, which can themselves be introduced using several different methods. A recent survey and comparison of various click partners found that the use of *endo*-bicyclononyne and methyltetrazine partners in an inverse electron demand Diels–Alder (IEDDA) cycloaddition was most effective (*k*_2_ = 70 M^–1^ s^–1^) and therefore these partners might be suitable for the first iteration of any click-mediated strategy.^21^

Two other general approaches have arisen to solve this problem, both of which aim to effectively increase the local concentration of the protein coupling partners. The first is to use two proteins of opposing charge (i.e., isoelectric points (pIs) either side of 7, Figure 3) in order to bring them into contact via electrostatic interactions.^22^ The second involves producing proteins with an affinity for a surface; this was achieved with a protein bearing a His_6_-tag which binds to an agarose surface displaying Ni(II).^23^ Although this approach was actually used for coupling of proteins to a liposome bearing Gly_3_ motifs, the concept should be applicable to protein–protein conjugation.

Despite the protein–protein coupling problem, several strategies have emerged for preparing protein–protein conjugates which are summarized herein. Methods encountered rely on chemical modification of native side chains (Section 3), incorporation of unnatural amino acids (Section 4), or the use of enzymatic reactions or sequence tags (Section 5).

## 3 Targeting Canonical Amino Acids

Attempts to generate protein–protein conjugates using chemical conjugation strategies have been pursued for over half a century.^24^ Early methods using bifunctional chemical reagents relied heavily on the inherent nucleophilicity of cysteine residues. Approaches included the use of reagents such as bifunctional cysteine-selective organomercury reagents to generate sulfur–mercury linkages between proteins.^25^ However, a more recognizable cysteine targeting strategy, which remains extremely popular to this day, is homobifunctional bismaleimide reagents, used for the conjugation of proteins through reduced cysteine thiols.^26^

Amino acids that contain nucleophilic amine or hydroxyl side chains were also exploited in early protein–protein conjugation strategies.^24^ Amine reactive bifunctional reagents incorporating functionalities such as diisocyanates, α,ω-dialdehydes including glutaraldehyde, and halonitrobenzenes, which also react with histidine and the hydroxyl groups of tyrosine, have been exploited in protein–protein conjugation.^27−29^ In addition, carbodiimides were used to cross-link carboxylic acids and free amino groups of different protein domains, while imidoesters were used to cross-link amine groups, including those found in lysine residues.^30−32^

A range of less residue specific, general nucleophile targeting, cross-linking strategies were also developed to generate protein–protein conjugates. These include bisepoxide, *s*-triazine, and aziridine functionalities and are discussed in a comprehensive review on cross-linking strategies.^24^

### 3.1 Cysteine-Targeting Reagents

Although many of the early strategies that targeted native residues successfully produced the desired protein–protein conjugates, they lacked specificity, resulting in conjugation through multiple residues on each protein. This drawback, coupled with the advent of powerful genetic engineering technology in the 1990s, meant interest in chemical generation of protein–protein conjugates did not endure. However, recent advances in both site-selective and site-specific protein modification strategies that exhibit exquisite control have prompted a resurgence in the pursuit of chemically linked protein–protein conjugates. There remains an overwhelming preference for targeting nucleophilic amino acid residues, in particular cysteine, due to its highly selective reactivity profile and low natural abundance.^33,34^

#### 3.1.1 Single-Residue-Targeting Homobifunctional Reagents

**Figure 2:**
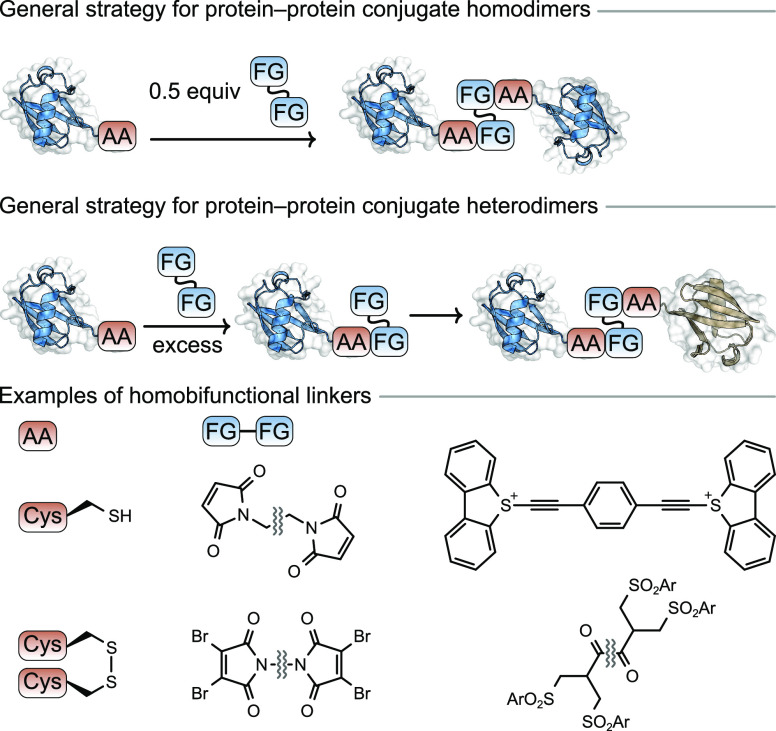
Strategies for preparing protein–protein conjugates using homobifunctional linkers.

Homobifunctional linking strategies rely on symmetric molecules with an identical reactive functionality on both ends of a linker. These homobifunctional molecules target the same amino acid residue on each of the protein domains being conjugated, although the specific environment does not necessarily have to be identical. These can be used to generate homo- or heterodimeric protein–protein conjugates in one pot or sequential reaction protocols, respectively (Figure 2).

A popular homobifunctional linking approach exploits one of the most ubiquitous conjugation strategies used in chemical biology; cysteine-maleimide conjugation (Figure 2).^22,35−38^ To achieve conjugation, an odd number of exposed cysteine residues are required to allow one end of the homobifunctional molecule to remain unconjugated and present a reactive handle to which a second protein can be conjugated. Conjugation of mouse monoclonal and rabbit polyclonal hinge cysteine-containing antigen binding fragments (Fab′), both containing three cysteine residues in their hinge region, was performed with an *ortho*-phenylenedimaleimide linker.^35^ This method was also used to prepare mouse–mouse and mouse–rabbit Fab′ bispecific antibodies upon addition of a second cysteine-containing Fab′. However, the even number of disulfide bonds found in the hinge region of human immunoglobulin G antibodies (IgGs) makes this approach incompatible with human Fab′ dimerization and therefore less therapeutically relevant.^35^

A variation of this approach used antigen binding fragment (Fab) re-engineering to introduce a reactive unpaired cysteine into a Fab and followed by dimerization with homobifunctional bismaleimide reagents.^36^ To achieve this, recombinantly expressed Fabs containing engineered cysteine residues, termed thio-Fabs, were conjugated using a bismaleimide coupling reagent with a polyethylene glycol (PEG) linker to form a bis-thio-Fab heterodimeric species named biFabs. Heterodimerization was achieved via the addition of an excess of bismaleimide to the initial thio-Fab, generating thio-Fabs presenting electrophilic maleimide handles. These could subsequently undergo conjugation to a second thio-Fab domain.

This particular study focused on thio-Fabs generated from the human epidermal growth factor receptor 2 (HER2)-targeting antibody, trastuzumab. All thio-Fabs conjugated in this manner varied only with respect to the location of the engineered cysteine in the Fab domain. All biFabs were therefore by definition monospecific, but could elicit extremely different biological responses. Depending on the orientation of the variable fragment (Fv) regions of the biFabs, the conjugates could either promote or inhibit breast tumor cell growth.^36^ This study highlights the impact that chemically conjugating protein domains at predefined internal sites without relying on N-to-C terminal ligation can have on biological properties.

**Figure 3:**
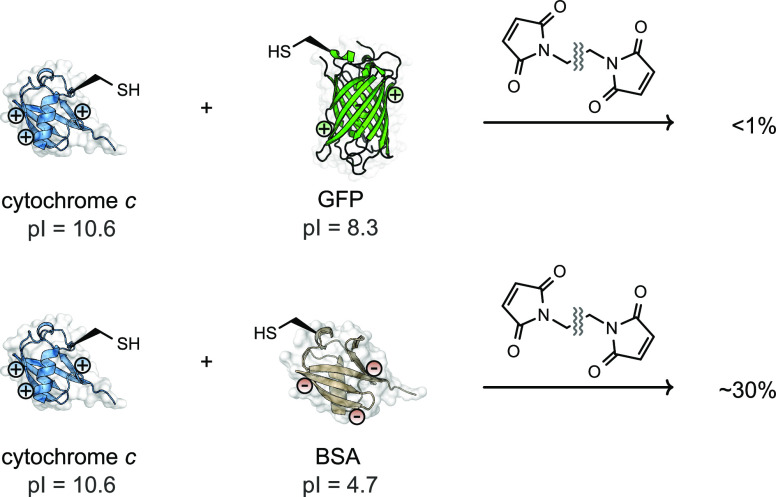
Role of opposing charges in preparation of protein–protein conjugates.

Beyond bispecific antibody production, homobifunctional bismaleimide reagents were used to explore the effect of global protein charge in the one-pot dimerization of high molecular weight proteins.^22^ Treatment of two proteins of opposing net charges (bovine serum albumin (BSA), pI = 4.7 and cytochrome *c*, pI = 10.6) with a bismaleimide reagent provided the corresponding heterodimer in yields of up to 30% (Figure 3). In contrast, two proteins of similar charges (cytochrome *c*, pI = 10.6 and GFP, pI = 8.3) under the same conditions gave the corresponding heteroconjugate in <1% yield, clearly demonstrating the importance of the physicochemical properties of precursors for protein–protein conjugation.

Although fast and accessible, maleimide conjugation strategies suffer from well-documented drawbacks, in particular the susceptibility of conjugates to undergo retro-Michael addition under physiological conditions.^37,39^ This characteristic is suboptimal for biologics, and therefore, alternative conjugation approaches exhibiting enhanced stability under physiological conditions have been the subject of much investigation. One such example is the use of *S*-alkynyl sulfonium reagents, which generate stable ubiquitin–ubiquitin homodimers (Figure 2).^40^

#### 3.1.2 Disulfide Rebridging Homobifunctional Reagents

Homobifunctional reagents based on rebridging of disulfide bonds in Fab and single chain variable fragment (scFv) antibody domains have also been employed to generate bispecific antibodies. These techniques are an accessible method for generating antibody dimers, as the constituent scFv domains can be easily acquired.

The development of a reagent that features two bis-sulfone groups for disulfide rebridging at either end of a PEG linker led to the preparation of Fab-PEG-Fab conjugates with comparable or better binding and in vitro efficacy than their corresponding parent IgGs, which target HER2 and vascular endothelial growth factor (VEGF) (Figure 2).^41^ This method was limited to the generation of homodimeric conjugates, and relatively low yields were achieved (18%). However, it was reported that the resulting dimers maintained their activity after storage at 4 °C for six months, highlighting the stability of the linkage generated by this disulfide rebridging approach.

Heterodimeric Fab-scFv conjugates were prepared using “next-generation maleimide” reagents that feature halogens on the *sp*^2^ carbons of the maleimide functional group. In this case, the reagent featured two 2,3-dibromomaleimide (DBM) reactive groups at either end of a PEG linker and was employed in an analogous manner to the bis-sulfone approach described previously (Figure 2).^42^ Yields of up to 52% were achieved in the production of heterodimeric conjugates using a sequential addition strategy. This strategy was subsequently improved using a more reactive and hydrolytically stable 2,3-diiodomaleimide (DIM) species.^43^ Exploiting the slower rate of DIM hydrolysis compared to DBM allowed more sterically hindered systems such as trimeric scFv formats and human serum albumin (HSA)-scFv or Fab conjugates to be produced. This was achieved by overcoming the competing hydrolysis of DBM to unreactive dibromomaleamic acid, allowing more sterically hindered thiols to react.^42,43^ Upon hydrolysis of DIM, serum stable maleamic acid conjugates were generated. However, incubation at 37 °C for up to 72 h was required for complete hydrolysis. Therefore, the development of conjugation strategies which directly form stable products, without the need for hydrolysis, may be beneficial to avoid extended incubation times.^43^

In general, homobifunctional disulfide linking approaches are advantageous because the corresponding dimers can be generated from any Fab which can be produced enzymatically from commercially available therapeutic antibodies. Conceptually, any disulfide rebridging reagent that can be placed at either end of a linker could be utilized to achieve similar effects to those described.^44^ This approach is therefore accessible to researchers without facilities for protein engineering and expression.

#### 3.1.3 Click Handle Installation at Cysteine

As one of the most ubiquitously exploited classes of bioorthogonal reactions, click chemistry has been widely utilized to generate protein–protein conjugates. The most commonly used reactions include those between terminal or strained alkynes with azides or tetrazines, in the presence or absence of Cu(I), depending on the specific reactive partners chosen.^45^ Once a bioorthogonal pair of components for click chemistry has been selected, they can be installed on proteins using one of several bioconjugation strategies.

**Figure 4:**
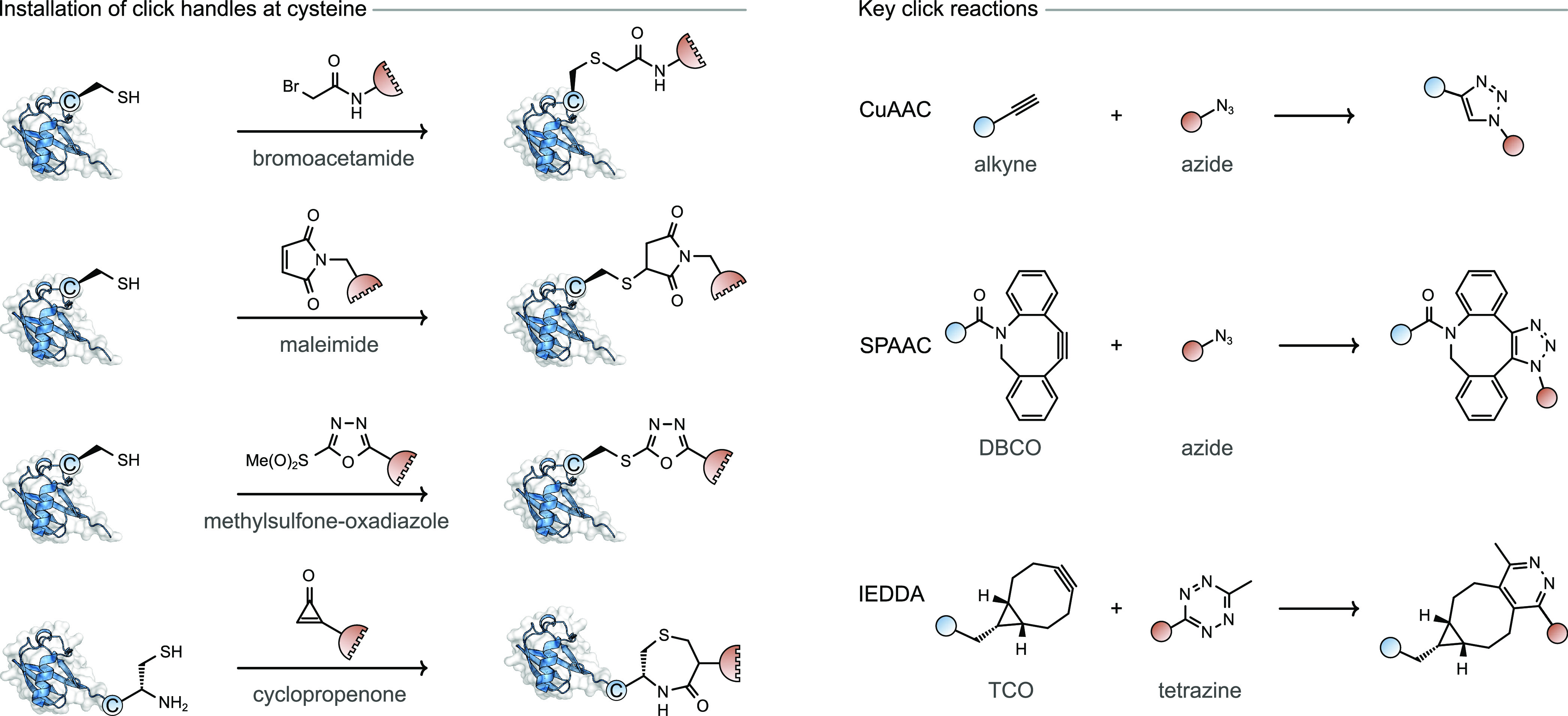
Production of protetin–protein conjugates by installing click groups on single cysteine residues.

Early attempts to generate protein–protein conjugates via click-based methods used Cu(I)-catalyzed azide–alkyne cycloaddition (CuAAC) between proteins bearing these two functionalities (Figure 4).^46−50^ One example of generating protein–protein conjugates in this way was to install the alkyne and azide groups at cysteine residues via bromoacetamide conjugation, generating di-scFvs upon dimerization via CuAAC.^46^ After conjugation of a reagent featuring a terminal trialkyne moiety in place of a monoalkyne derivative, an improvement in conversion from 33% to 74% was observed and this was attributed to an increased effective concentration of alkyne. The binding to the Mucin-1 peptide, prostate, and breast cancer cell lines was up to four times higher for the dimers compared to the parent scFv fragments. Similar results were also observed in subsequent CuAAC-mediated conjugation of di-scFvs, successfully generating multivalent conjugates.^47^ Approaches using CuAAC were also used to produce cross-linked hemoglobin^48,49^ and BSA-lipase heterodimers.^50^

Alternative click-based methods which do not require Cu(I) catalysis have been explored, including strain-promoted azide–alkyne cycloaddition (SPAAC) and strain-promoted IEDDA cycloaddition in which strained unsaturated systems such as dibenzocyclooctyne (DBCO), *trans*-cycloctene (TCO), or bicyclo[6.1.0]nonyne (BCN) react with either azide or tetrazine moieties (Figure 4).^45^ In the case of SPAAC conjugation, an azide undergoes a click reaction with a strained alkyne such as DBCO, to generate protein–protein conjugates.^13,51−53^

SPAAC conjugation enabled the dimerization of antiprostate-specific membrane antigen (PSMA) and anti-cluster of differentiation 3 (CD3) Fab fragments to generate bispecific T cell engaging antibodies against prostate cancer.^51^ DBCO handles were installed on Fab fragments via a disulfide reduction and rebridging approach with heterofunctional dibromomaleimide molecules, in a conceptually analogous method to the aforementioned disulfide rebridging homobifunctional linking strategies.^42^ Subsequently, a PEG bis-azide reagent was used to introduce a surface exposed azide moiety which could in turn react with a second DBCO-Fab fragment to generate bispecific antibodies. The bispecific antibody produced using this method displayed high potency in the picomolar range against PSMA-expressing prostate cancer cell lines and selectively bound the respective antigens of the constituent Fab fragments (PSMA and CD3), indicating that the conditions used in SPAAC conjugation were sufficiently mild to conserve the biological activity of the parent domains. This approach was also demonstrated on full length IgGs by installing azide and DBCO handles in the hinge region of anti-HER2 and antiepidermal growth factor receptor (EGFR) antibodies to generate a full length bispecific antibody which retained the potency of its constituent domains.^52^ Another study installed azide and DBCO handles, using bromoacetamide reagents to introduce these click partners at reduced disulfide cysteine residues in the hinge region of anti-CD3 and anticarcinoembryonic antigen (CEA) antibodies.^53^ This approach generated bispecific T cell engagers (BiTEs) based on full length antibodies termed, dual-specific, bivalent BiTEs. These dual-specific, bivalent BiTEs were shown to successfully redirect T cells to kill CEA^+^ cells in transgenic mice. Use of cyclopropenone-based reagents, which react selectively with N-terminal cysteine residues over other internal cysteine residues, were used to produce N-to-N terminally conjugated dimers of a de novo designed mimic of the IL2 cytokine.^54^

IEDDA click chemistry is another tool available for generating protein–protein conjugates typically employing the strained molecules TCO or BCN, in conjunction with tetrazine moieties. Using this click chemistry approach, dimerization of T4 lysozyme resulted in an 8-fold improvement in yield compared to a bismaleimide homobifunctional strategy, with yields of 38% and 5%, respectively.^55^

A homobifunctional click reagent featuring potassium acyltrifluoroborate (KAT) groups connected by a PEG linker was used to generate homodimers of cysteine-containing T4 lysozyme and superfolder GFP (sfGFP) mutants.^56^ The latter conjugation strategy introduced hydroxylamine functional groups at engineered surface exposed cysteine residues using cysteine-selective bifunctional molecules featuring a methylsulfonephenyl-oxadiazole-hydroxylamine motif. The hydroxylamine functionalized proteins were found to rapidly react with homobifunctional KAT reagents, giving up to 72% conversion after 5 h. However, it should be noted that the reaction was found to proceed most efficiently at pH 3.6. The necessity for highly acidic conditions could make this approach less compatible with sensitive protein domains.

**Figure 5:**
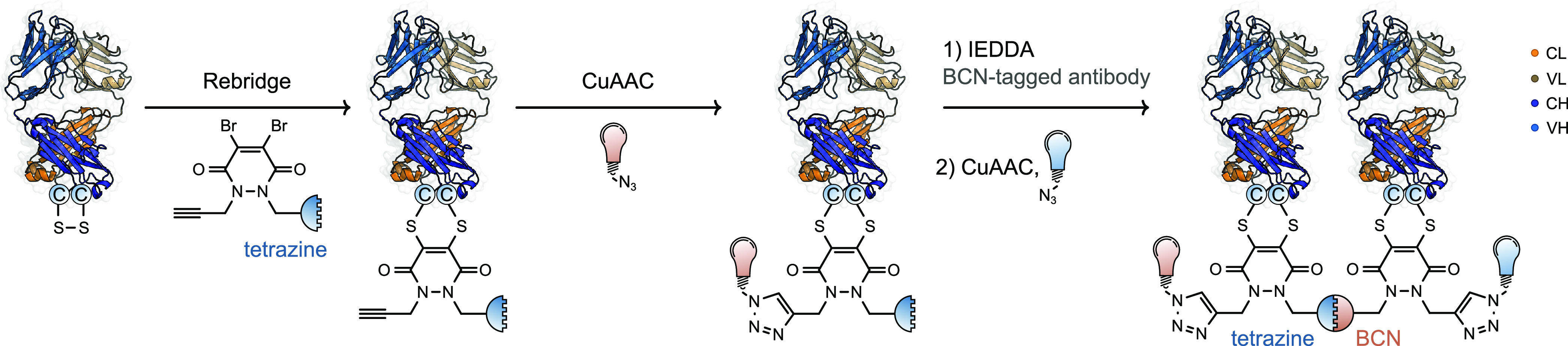
Production of protein–protein–dye conjugates using orthogonal click chemistry and disulfide rebridging.

The orthogonal nature of IEDDA and CuAAC click chemistry was exploited to generate bispecific antibodies which were subsequently dual-functionalized with two different payloads (Figure 5).^57^ Disulfide rebridging of two Fab fragments was carried out with bifunctional pyridazinedione reagents functionalized with either the strained alkyne BCN or a tetrazine moiety, respectively. These two orthogonally labeled conjugates were subsequently coupled using an IEDDA reaction to generate bivalent and bispecific antibodies from trastuzumab, rituximab, and cetuximab, which target HER2, CD20, and EGFR receptors, respectively. Since both nitrogen atoms of the pyridazinedione can be functionalized, a second orthogonally reactive terminal alkyne moiety was introduced. Upon generation of the conjugates, CuAAC chemistry was carried out to generate bispecific antibodies labeled with Alexa Flour 488 in 56% yield. By prelabeling the tetrazine-bearing fragment with azide-bearing sulfo-Cy5.5 dye, a dual-labeled antibody was generated by ligating a second dye, in the form of an azide-bearing Alexa Flour 488, after bispecific antibody generation with an impressive yield of 55%. In doing so, the authors demonstrated the potential for labeling of chemically generated bispecific antibodies with well-defined conjugation patterns of one, two of the same, or two unique payloads. Although not explored in this study, this approach could in theory be extended to drug payloads, thus producing a dual payload bispecific antibody–drug conjugate.^58^

#### 3.1.4 Cysteine Reactive Heterobifunctional Reagents

An alternative class of compounds used in protein–protein conjugation are heterobifunctional reagents. These comprise a small molecule reagent containing two orthogonally reactive moieties connected by a linker. Due to the challenges associated with targeting two distinct amino acid residues on separate protein domains, while avoiding intramolecular cross-linking, this approach is challenging to execute. However, a number research groups have managed to overcome these challenges using orthogonally reactive functionalities with high specificity.^59−61^

One approach exploited with heterobifunctional reagents is to protect one reactive functionality during the first conjugation step. Using a bis-sulfone functionality, discussed in Section 3.1.2, combined with a maleimide functionality, an orthogonal cysteine-selective heterobifunctional reagent controlled by a pH switch was developed.^59^ At pH 6, upon addition of an excess of heterobifunctional reagent, the maleimide functional group is sufficiently reactive to undergo conjugation at cysteine on the first protein domain. After dialysis and increasing the pH to between 7–8, the bis-sulfone functional group underwent E1cB elimination of *p*-toluene sulfinic acid thus generating a reactive Michael acceptor moiety on the protein which reacted with the second cysteine-containing protein. This enabled the preparation of HSA–BSA heterodimers in 10% yield. Although pH switching is an interesting concept, an analogous product might have been achieved with a bismaleimide conjugation strategy without the need for pH switching.

**Figure 6:**
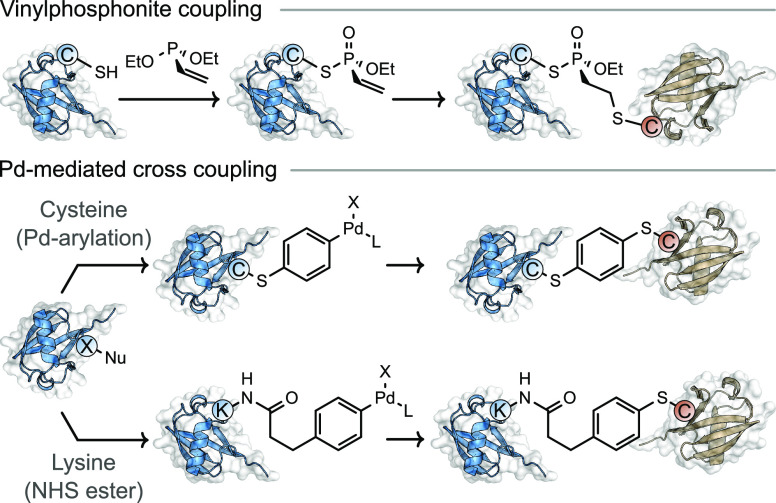
Use of vinylphosphonite and Pd-protein OAC heterobifunctional reagents for sequential protein conjugation through cysteine residues.

Another strategy using heterobifunctional molecules relies upon the activation of a functional group toward a second conjugation step only after it has undergone reaction with the first protein domain. In one example, a vinylphosphonite reagent was used to generate an electrophilic, cysteine-reactive, vinylphosphonothiolate handle at a modified cysteine (Figure 6).^60^ This strategy was demonstrated by producing diubiquitin and ubiquitin−α-synuclein conjugates with up to 80% conversion. However, the scope beyond the generation of relatively small ubiquitin-containing conjugates, a field which has been widely studied, has not yet been demonstrated.^62^

In addition to solely targeting a single cysteine residue, reactive functionality pairings such as *N*-hydroxysuccinimide (NHS)-ester/maleimide or *N*-[ϵ-maleimidocaproyloxy]sulfosuccinimide ester (Sulfo-EMCS)/maleimide that target lysine and cysteine residues, respectively, were developed.^61^ These approaches successfully generated protein–protein conjugates; however, multimeric species were also produced due to the nonspecific nature of targeting lysine residues.^63^ Nonetheless, this relatively crude linking strategy allowed researchers to investigate the effect of chemically linked *Plasmodium falciparum* Merozoite Surface Protein-1 conjugates on immunogenicity in mice when compared to monomeric or oligomeric forms.^61^ This study highlights that, occasionally, heterogeneous protein–protein conjugates can be used to answer biological questions without resorting to more complex conjugation strategies.

#### 3.1.5 Metal-Mediated Conjugation Strategies

In the field of site-selective protein modification, metal-mediated cross-coupling reactions on protein substrates are rapidly gaining interest. A range of metal-mediated, cysteine selective, coupling strategies using Pd(II), Au(I/III), Ni(II), and Pt(II)-based organometallic reagents have been developed for the chemoselective modification of cysteine.^64−69^ A variety of metal-mediated methods have subsequently been used to generate protein–protein conjugates, typically employing a heterobifunctional linking approach.

Of the metal-based conjugation strategies, novel methods used to generate bench stable Pd-protein oxidative addition complexes (Pd-protein OACs) have received the most interest for producing protein–protein conjugates.^70,71^ Initial studies site-selectively modified cysteine using a Pd-OAC generated from 1,4-dihaloarene, proceeding through a hypothesized π-complex intermediate followed by intramolecular oxidative addition to generate the aforementioned Pd-protein OAC (Figure 6).^70^ Upon formation of the Pd-protein OAC, the electrophilic handle installed at cysteine subsequently reacted with a solvent exposed cysteine residue on a second protein, effectively generating heterodimeric conjugates through the formation of stable C(*sp*^2^)–S bonds. This method is conceptually similar to the previously discussed vinylphosphonite reagent which undergoes sequential activation after reacting with a cysteine residue on the first protein domain.^60^ Conversions of up to 79% were successfully achieved, and the method was demonstrated on proteins with molecular weights of up to 83 kDa, clearly displaying the general applicability of this method for cysteine-containing proteins of various sizes.

This approach was applied to smaller synthetic proteins derived from flow peptide synthesis (<100 amino acids).^72^ By combining flow synthesis and Pd-mediated cysteine–cysteine conjugation chemistry, a panel of bioactive, covalently cross-linked transcription factor (TF) homo- and heterodimers were generated. These displayed improved stability compared to the corresponding noncovalent complexes and led to inhibition of oncogenic proliferation. This study clearly demonstrates the potential that purely synthetic methods hold in developing active bimolecular protein therapeutics. Interestingly, the same homo- and heterodimeric TFs were produced using a solely automated flow approach developed in parallel by the same group.^73^ Although this approach produced similarly bioactive TF homo- and heterodimers which also attenuated oncogenic activity, this method resulted in low yields (14%) when compared to other chemical conjugation approaches. Nonetheless, this research highlights the potential that flow synthesis holds for rapid, high-throughput production of protein–protein conjugates, and over time will undoubtedly be further optimized to achieve improved yields.

Protein–protein conjugates were prepared through lysine residues using a heterobifunctional molecule comprising both an amine-reactive NHS ester and a Pd-OAC functional group (Figure 6). Initial acylation of lysine residues with the NHS ester was used to install a Pd-OAC group, which could subsequently undergo a second conjugation step with the cysteine residue of a different protein.^71^ One downside was the unselective nature of the NHS ester acylation strategy making this approach less useful for generating well-defined protein–protein conjugates. This was demonstrated through the generation of a heterogeneous mixture of RNase A-Pd OAC complexes.^71^ Nonetheless, this approach was effectively used to generate protein–protein conjugates with high yields and was even shown to proceed at nanomolar concentrations, highlighting the fast reaction kinetics of the Pd-Protein OACs.

In addition to Pd-mediated conjugation, proteins were also conjugated via cysteine arylation chemistry using bis-arylboronic acid homobifunctional polymers and a Ni(II) catalyst.^67,74^ This approach was used to produce GFP and T4 lysozyme homodimers with conversions of approximately 50%.^74^ In addition, Cu(II)-catalyzed, histidine directed, backbone N–H arylation and alkenylation of proteins with boronic acids was achieved.^75^ By sequential addition of a heterobifunctional linker featuring 2-nitro-arylboronic acid and (*E*)-alkenylboronic acid functionalities, orthogonal Ni(II)-promoted cysteine arylation followed by Cu(II)-catalyzed histidine-directed backbone N–H alkenylation was achieved.^76^ This strategy allowed heterodimeric protein–protein conjugates of T4 lysozyme and sfGFP to be generated with up to 93% conversion.

As with all approaches utilizing organometallic compounds, there remains the issue of complete removal of metal ions which may chelate to proteins altering their function or causing downstream toxicity. It was noted in the case of Pd-protein OACs that only 90% of the Pd, as determined by inductively coupled plasma mass spectrometry, was removed from the purified conjugates.^70^ For metal-mediated protein–protein conjugation to find relevance in the generation of therapeutics, issues with potential metal-mediated toxicity and their complete removal require addressing.

### 3.2 Lysine Targeting Reagents

Reagents targeting the ϵ-amine of lysine residues are a popular approach to generate bispecific antibodies. The methods are simple to execute and have therefore been widely used, but suffer from issues including unselective labeling at multiple lysine residues which can lead to sample and batch heterogeneity.

**Figure 7:**
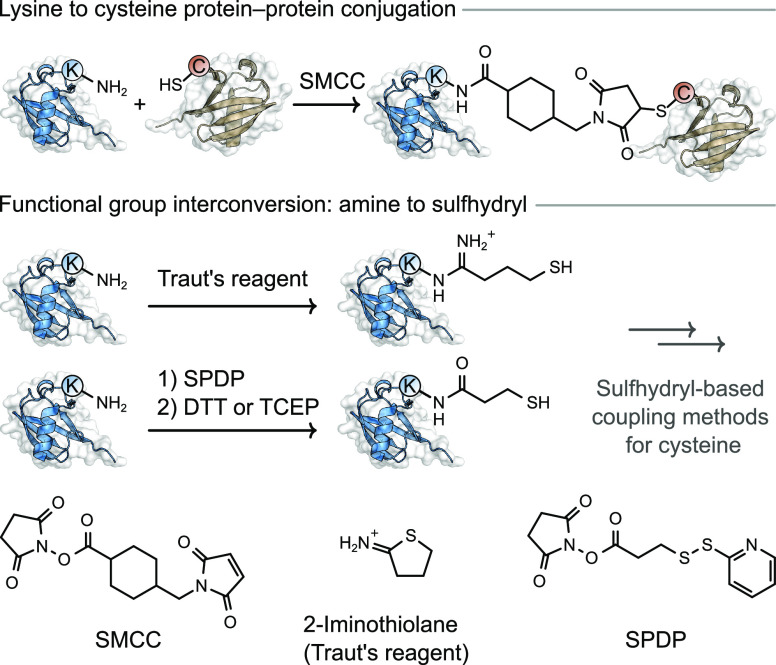
Methods for preparing protein–protein conjugates using lysine conjugation. DTT: Dithiothreitol. TCEP: tris(2-carboxyethyl)phosphine.

A popular approach involves an effective functional group interconversion from a primary amine (lysine) to a thiol. This can be achieved with *N*-hydroxysuccinimide-succinimidyl-3-(2-pyridylthiol)propionate (SPDP), generating solvent exposed thiols on both antibodies after a reduction step (Figure 7).^77,78^ This chemistry was applied to two separate antibody domains, and the resulting sulfhydryl-containing proteins were incubated together in a 1:1 ratio to generate bispecific conjugates linked by a disulfide bond. This strategy was used on multiple occasions to develop a range of bispecific antibodies with uses in imaging and as a potential therapeutic for autoimmune thyroiditis.^79,80^ This method suffers from the lack of selectivity for homo- versus heterodimerization, resulting in a statistical mixture of products. It is also notable that disulfide bonds formed in bioconjugation can be unstable under physiological conditions, making these conjugates less useful as therapeutic agents.^81,82^

Alternative approaches for selectively targeting lysine residues that overcome some of the challenges faced when using SPDP avoided the requirement for disulfide bond formation altogether. The cyclic compound thioimidate 2-iminothiolane, commonly known as Traut’s reagent, has been used to introduce a free thiol at lysine, which can subsequently react with a maleimide functional group installed at lysine on a second protein using bifunctional succinimidyl 4-(*N*-maleimidomethyl)cyclohexane-1-carboxylate (SMCC) reagents (Figure 7). Bispecific antibodies capable of simultaneously directing stem cells to infarcted myocardium and T cells to tumors were generated using this method.^83,84^ This linking strategy, combining Traut’s reagent and SMCC reagents, was also used to generate therapeutic bispecific antibodies in other preclinical cancer studies.^85,86^ Nonetheless, this approach suffers from the previously discussed retro-Michael addition and thiol exchange problem faced by all maleimide–thiol conjugation strategies.^37,39^

A further lysine-selective method designed to overcome stability issues arising from disulfide or maleimide linkages was recently described. Benzaldehyde and hydrazine functional groups were introduced using NHS ester conjugation at lysine, and the resulting proteins were mixed in a 1:1 ratio, generating a hydrazone-linked species.^87^ This approach was used to generate T cell recruiting bispecific antibodies to cancer cells overexpressing EGFR. However, it should be noted that hydrazones are not completely stable under aqueous conditions and can undergo hydrolysis.^88^

### 3.3 Reagents Targeting Alternative Amino Acid Residues

Targeting amino acids beyond cysteine and lysine when generating protein–protein conjugates is currently an underdeveloped area. This is mainly due to the high selectivity achieved using cysteine-targeting reagents that is rarely possible with other canonical amino acids.

One approach which achieved protein–protein conjugation through alternative nucleophilic residues utilized a photocaged quinone methide functional group linked to an NHS-ester.^89^ This approach installed the photocaged quinone functional group onto a protein at a lysine residue via amine acylation. Subsequent UV irradiation generated a highly reactive Michael acceptor in the form of a quinone methide. This intermediate was trapped with any of nine amino acid residues on a second protein domain: Asp, Glu, Lys, Ser, Thr, Tyr, Gln, Arg, and Asn, with Gln, Arg, and Asn being of particular interest as they have rarely been probed using other cross-linking approaches. This method was used to covalently cross-link proteins in vitro and generate protein–DNA cross-links. Although the objective of this research was to study biomolecular interactions through cross-linking and not to generate well-defined conjugates, the promiscuous nature of this approach clearly highlights the challenges that could arise when attempting to generate protein–protein conjugates through alternative amino acid residues.

## 4 Genetic Code Expansion

Protein–protein conjugates have also been prepared by incorporating unnatural amino acids (UAAs) via codon reallocation. Referred to as genetic code expansion (GCE), this process takes advantage of endogenous protein synthesis machinery to incorporate a reactive handle, such as an azide, which is chemically distinct from the functional groups of the 20 canonical amino acids.^90^ This enables the use of click chemistry for site-specific protein modification and leads to products with excellent homogeneity. This selectivity, coupled with the diversity provided by over 200 reported UAAs, has led to the utilization of GCE, with a particular focus on bispecific antibodies.^91^ The most common GCE-mediated approach for preparing bispecific antibodies is to combine two UAA-containing proteins with a bifunctional reagent featuring a flexible linker. Linker lengths and conjugation sites can be readily optimized, and this modular approach is amenable to the combinatorial generation of broad heterodimer libraries.^92^

A well-studied UAA linking strategy involves the formation of an oxime followed by click chemistry. A notable example of this approach is the coupling of a *p*-acetylphenylalanine (pAcF) residue with an alkoxyamine bifunctional linker, via a oxime bond, to install either a terminal azide or an alkyne group into anti-HER2 Fabs. Once installed, the Fabs were conjugated with CuAAC. The affinity of the resulting anti-HER2 Fab homodimers was comparable to a full-length IgG and exhibited subnanomolar killing (EC_50_ ≈ 20 pM) of HER2^+^ cancer cells in the presence of human T cells in vitro.^92^ This methodology was expanded to generate higher valency IgG and Fab-based bispecific antibodies, including Tri-Fab, Tri-IgG, and Tetra-IgG conjugates, produced in yields of 30%, 25%, and 50%, respectively.^93^ Despite being comparable to the most potent bispecific formats, the need for low pH (4.5) and long reaction times (72 h) may restrict the generality of this approach, rendering it incompatible with sensitive protein domains.^92,94^

Other strategies utilized heterobifunctional linkers that react selectively with natural amino acids on one protein and unnatural amino acids on another. Such approaches are commonly based on site-selective cysteine chemistry, as highlighted by a aminooxy-maleimide reagent, which was used to conjugate a pAcF-containing Herceptin Fab to Sap 6 containing an engineered cysteine residue.^95,96^ However, strategies that are not based on cysteine conjugation also exist. For example, by combining an UAA with a *o*-methoxyphenol side chain, which undergoes site-specific oxidative coupling with an aniline functionality in the presence of NaIO_4_, with a N-terminal selective 2-pyridinecarboxaldehyde moiety, a well-defined dimer of RNase and the *p*-amino-l-phenylalanine-MS2 viral capsid was generated.^97^

**Figure 8:**
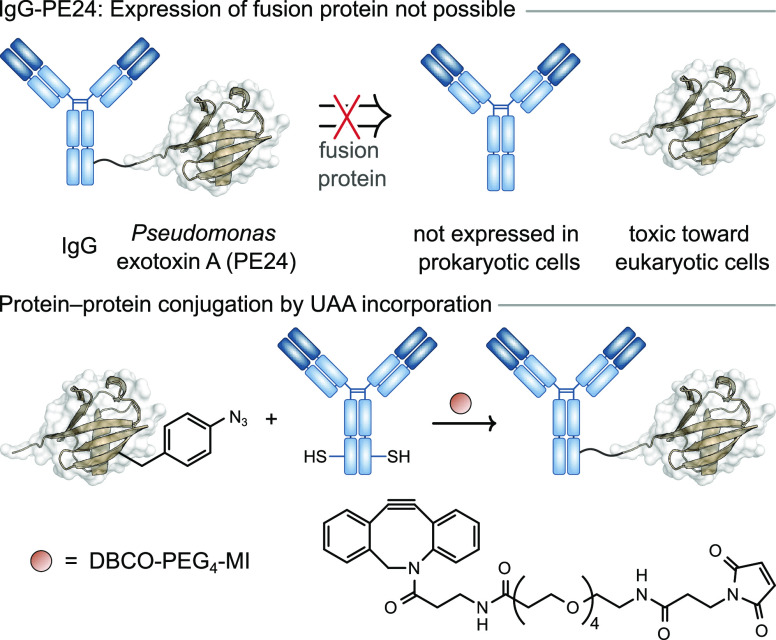
Incorporation of an unnatural amino acid (UAA) enables the preparation of IgG-PE24, a protein–protein heterodimer that is not accessible via a fusion protein pathway.

UAAs have also been deployed in the generation of full-length IgG immunotoxins (Figure 8).^13^ Immunotoxins are chimeric fusion proteins consisting of a cancer targeting antibody fragment and a bacterial protein toxin, which can be used to kill cancerous cells.^98^ For example, *Pseudomonas* exotoxin (PE), containing the UAA azidophenylalanine was expressed in bacteria and conjugated to a HER2 targeting IgG expressed in a mammalian system and functionalized with a DBCO handle via maleimide chemistry at an engineered cysteine residue.^13^ This approach generated immunotoxins with highly target specific cytotoxicity against HER2^+^ cell lines. Due to the inherent toxicity of PE to eukaryotic cell lines, production of full length IgG immunotoxins via traditional fusion methods has not been successful to date.^13^ The ability to independently express both IgG and PE domains in separate cell lines prior to chemical generation of immunotoxins clearly highlights the benefits of post-translational conjugation methods in generating otherwise inaccessible protein–protein conjugates. Although these methods achieved site-specific protein–protein conjugation, the reagents necessitated a two-step conjugation process; after the first conjugation reaction, excess reagents were removed by dialysis or affinity purification.^99,100^ This drawback led to the exploration of alternative GCE-based protein conjugation methods.

Inspired by examples found in nature, attempts have been made to prepare linker-free protein–protein conjugates, referred to as direct protein–protein conjugation.^101^ Given this strategy involves a single site modification, protein–protein conjugation can potentially be achieved in a single-pot reaction with minimal impact on protein structure and function. Initial efforts utilized *p*-propargyloxyphenylalanine and *p*-azidophenylalanine side chains, which were coupled via CuAAC. Despite achieving protein–protein conjugation, the application of this method was limited due to the requirement of a cell-free protein expression system which resulted in low protein yields and, in addition, Cu-induced protein damage.^101^ In response to these limitations, a high yielding conjugation method was developed that incorporated an azide-containing amino acid into one protein and a BCN-containing amino acid into the other. This allowed for two proteins, glutathione S-transferase and a maltose-binding protein, to be conjugated via a SPAAC reaction, requiring no additional reagents.^99^

Despite these advances, the most prominent drawback of UAA mediated protein–protein conjugation is the efficiency with which the UAA can be incorporated, and in particular how the sequence context surrounding the in-frame UAG codon can restrict or prevent incorporation at particular sites.^102^ However, new codon reassignment technologies, such as the one used to incorporate *N*^ϵ^-(*o*-azidobenzyloxycarbonyl)-l-lysine, are enabling the development of UAA-based protein–protein conjugation.^17,103^

## 5 Enzymatic Methods and Tag Engineering

Recent years have witnessed increased interest in enzymatic and tag-based methods for protein modification.^104,105^ Such methods offer mild conditions and excellent site-specificity as a result of the enzymes used, but do require larger “sequence tags”—a specific sequence of amino acids—to be incorporated via recombinant protein expression.

### 5.1 Sortase-Based Approaches

**Figure 9:**
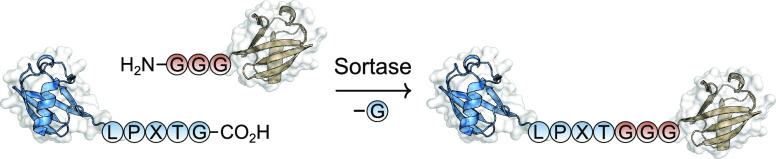
Sortase-based approach for preparing protein–protein conjugates.

One of the first examples using an enzymatic method to prepare a protein–protein conjugate employed sortase, a prokaryotic enzyme that catalyzes amide bond cleavage of a C-terminal LPXT↓G motif and transfers the remaining N-terminal polypeptide sequence to an appropriate nucleophile (Figure 9). Polyglycine sequences are privileged in their role as nucleophiles in this transpeptidation reaction, effectively enabling coupling between proteins bearing the sortase tag at the C-terminus and a polyglycine tag at the N-terminus of two protein coupling partners. In a proof-of-concept study, this method was used to prepare dimers of GFP.^106^

A sortase-mediated approach was used to couple the SRC Homology 3 (SH3) domain, which is insoluble at physiological pH, with the B1 domain of Protein G (GB1), thus endowing it with suitable solubility properties for structural characterization by NMR spectroscopy.^107^ The study also discovered that yields and reaction times were improved by performing the coupling under conditions of dialysis, a result arising from removal of the cleaved glycine-containing peptide from the reaction equilibrium.^107^

Sortase-based methods were also used to generate N-to-N and C-to-C linked protein–protein heterodimers, products that are impossible to generate using genetic approaches alone. This method relies upon the sortase-mediated installation of bioorthogonal click handles to either the N- or C-terminus, followed by SPAAC (Figure 11).^12^ This hybrid approach using sortase-mediated bioconjugation followed by click chemistry was used to prepare a library of heterodimeric protein conjugates based on the individual ligands neuregulin-1β (NRG) or epidermal growth factor (EGF).^108^ Each of the proteins intended for conjugation were functionalized with either a tetrazine handle at their C-terminus or a norbornene functional group at their N-terminus; subsequent mixing resulted in the formation of the desired heterodimers.^108^

A similar hybrid approach was used to prepare bispecific antibodies with broad anti-influenza virus activity.^109^ In this system, sortase-mediated conjugation was used to append DBCO and azide functional groups to the C-termini of two different IgGs. Interestingly, the addition of the DBCO functional group to the C-terminus was less efficient than the azide; this was suggested to arise from the promiscuous reaction of DBCO with free thiol groups that exist in both antibodies and sortase.^109^ Nonetheless, upon mixing of the orthogonally tagged coupling partners at 20 °C the desired bispecific antibody formed and displayed excellent stability, with >90% remaining after 3 weeks at 37 °C in IgG-depleted human serum.^109^ This chemo-enzymatic approach was also applied to the preparation of a bispecific antibody that recruits T cells to acute myeloid leukemia (AML) cells. An antibody and scFv domain were conjugated using sortase-mediated addition of tetrazine and TCO functional groups to the respective coupling partners.^110^

Building upon this hybrid approach, and using more recent knowledge that simple alkylamines can be substituted for the polyglycine motifs often used for sortase-mediated conjugation, protein–protein dimers and tetramers were produced.^111,112^ The conjugation strategy first employed the introduction of appropriate click handles (DBCO and azide, respectively) at the C-termini of the nanobody Ty1. Simple treatment of the bioorthogonally tagged proteins produced the Ty1–Ty1 homodimer, while the use of a tetra-azide reagent with excess DBCO-tagged Ty1 produced the homotetramer (Ty1)_4_. Because the Ty1 nanobody binds to and neutralizes the SARS-CoV-2 virus, producing the (Ty1)_4_ homotetramer achieved an IC_50_ value in the low picomolar range.^112^

Sortase-based methods have developed to the point where increasingly ambitious applications have started to emerge. A library of bispecific binding proteins was generated from two orthogonal sets of sublibraries, one of which comprised proteins bearing a sortase-tag followed by a His_6_-tag at their C-termini. A second library consisted of proteins bearing a Gly_5_-tag followed by a tobacco etch virus (TEV) protease cleavage site at their N-termini. Treatment of the combinatorial library with TEV protease for revealing the N-terminal Gly_5_-tag and sortase for protein–protein coupling led to the formation of the desired protein–protein heterodimers. Screening the library of bispecific antibodies led to the identification of both known (in protein fusion format) and unknown bispecifics that caused changes in cell proliferation in two cell lines of relevance to breast cancer.^113^

Sortase-based methods for preparing protein–protein conjugates are becoming some of the most widely applied, in particular for the addition of orthogonal functional groups that enable protein–protein conjugation via click chemistry. Recent studies have shown that the C-terminal LPXTG acceptor motif works for not only N-terminal polyglycine motifs and primary amines but also an engineered internal sequence (YKPH) which opens the door to nonlinear protein–protein conjugates using sortase-based methods.^114^ Finally, incorporation of a cysteine residue before the C-terminal sortase sequence tag enabled both protein–protein conjugation and protein–fluorophore conjugation.^115^

### 5.2 Approaches Mediated by Tyrosine Oxidation

**Figure 10:**
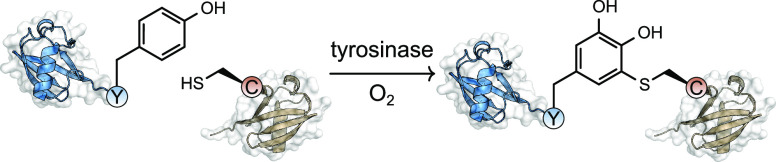
Tyrosine-based approached for protein–protein conjugation.

Recently, tyrosine has garnered interest as a target residue for producing protein–protein conjugates. In the presence of a tyrosinase, the phenol side chain is oxidized to an *ortho*-quinone functional group which forms the basis for further elaboration (Figure 10). A “knob-in-hole” antibody that features a G_4_Y motif at one of the C-termini was first oxidized with mushroom tyrosinase (mTyr) to provide an *ortho*-quinone group that can undergo a strain-promoted cycloaddition with BCN-functionalized proteins, including the cytokine IL2 or a short-chain variable fragment (scFv).^116^ The BCN-functionalized coupling partners were themselves prepared using a sortase-mediated conjugation, highlighting the potential of combining conjugation strategies to provide protein–protein conjugates.

The C-terminal Y-tag G_4_Y has also been targeted with other oxidizing enzymes for protein–protein conjugation.^117^ Treatment with enzymes such as laccase or horseradish peroxidase in conjunction with H_2_O_2_ can lead to the formation of protein–protein heterodimers including an IgG partner, although higher order oligomeric species have also been observed.^118−121^

Later work discovered that the *ortho*-quinone functional group generated upon tyrosine oxidation undergoes a reaction with the sulfhydryl group of a free cysteine residue.^122,123^ This approach was used to prepare conjugates of sfGFP with three different proteins: CRISPR-Cas9, a HER2-binding scFv, and nanoluciferase.^122^ Further work explored the use of different tyrosinases to expand the scope of tyrosine residues that could be targeted in this manner.^19^ A tyrosinase from *Bacillus megaterium* (megaTYR) was found to be more promiscuous and enabled the oxidation of tyrosine residues in a high number of sequence motifs, as assayed by peptide experiments. Further engineering of megaTYR led to a variant that displayed high activity toward tyrosine residues in the E_4_Y motif, ultimately enabling the construction of a linear triple-protein conjugate comprising nanoluciferase, GFP, and mCherry. One limitation of this approach, namely direct residue-to-residue conjugation, is that it does not present an opportunity for a longer linker between two proteins. Such linkers are easily achieved using traditional linker-based approaches, which can be obtained using the chemical cross-linking strategies described in Section 3. This limitation could become problematic when optimizing the binding of a bispecific antibody to an antigen.

**Figure 11:**
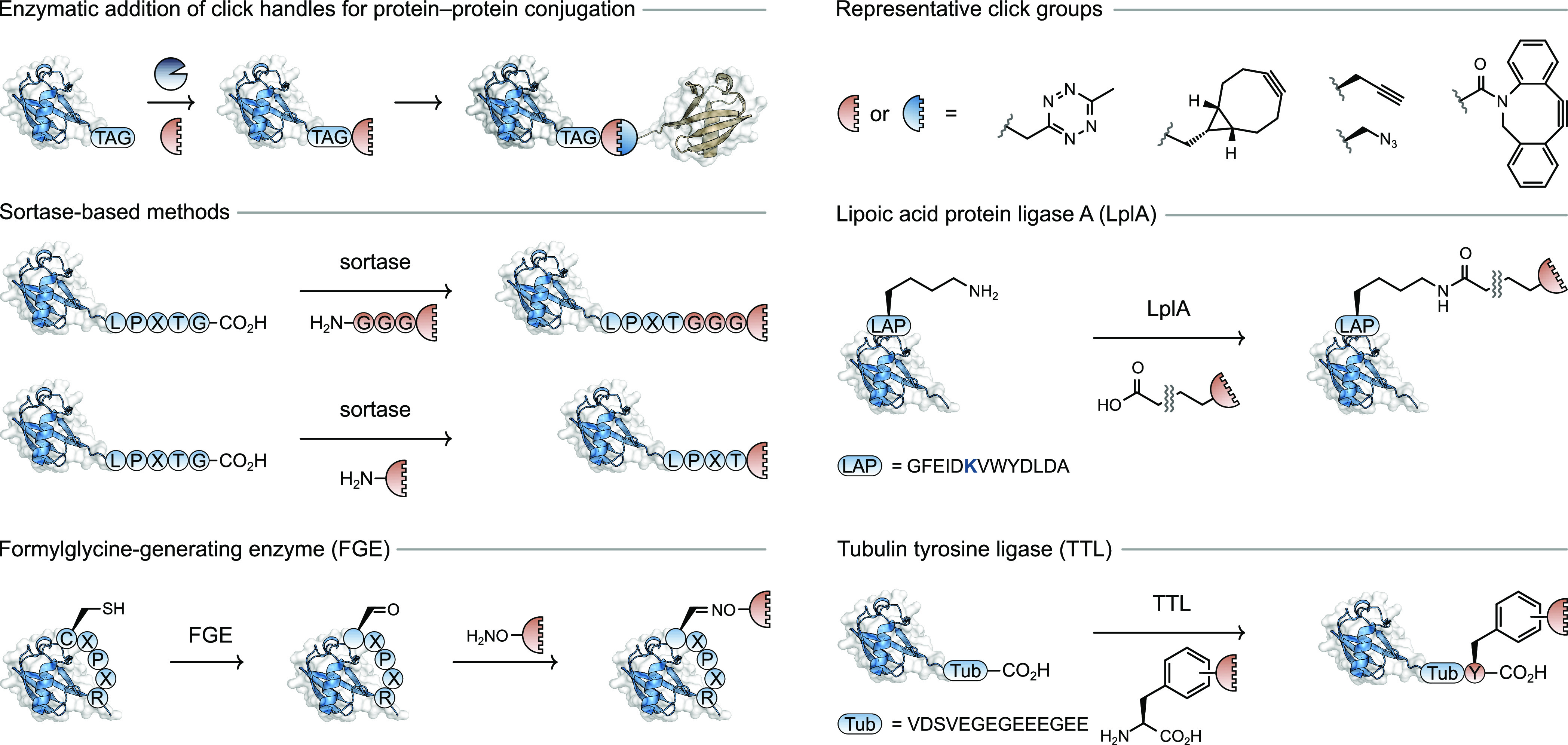
Enzymatic approaches to install click chemistry functional groups for protein–protein conjugation.

The enzyme tubulin tyrosine ligase (TTL) appends a tyrosine residue to an α-tubulin derived C-terminal recognition sequence (Tub). In a process conceptually related to metabolic engineering, a protein of interest bearing the Tub sequence was exposed to an analogue of tyrosine that contained either an azide or alkyne group on the phenyl ring (Figure 11).^124^ In the presence of TTL, the modified tyrosine was ligated to the C-terminus, producing a protein with a bioorthogonal handle that could be used in a subsequent protein–protein conjugation step using CuAAC. This procedure was used to prepare a protein–protein homodimer of the GFP-binding protein (GBP) in ∼50% conversion after 90 min. The coupling of a trastuzumab derived single chain variable fragment (TscFv) and GBP produced a protein–protein heterodimer in 62% yield after purification by size exclusion chromatography (SEC). Fluorescence microscopy showed that this heterodimer could recruit GFP to the plasma membrane of cells overexpressing the HER2 receptor, a target of TscFv.

### 5.3 Other Examples

Recently, a new enzymatic method emerged for protein–protein conjugation that requires a minimal sequence tag for enzyme recognition (IKXE). The E2 small ubiquitin-like modifier (SUMO)-conjugating enzyme, Ubc9, catalyzes the formation of an isopeptide bond between the lysine residue in the recognition sequence and a protein bearing a C-terminal thioester. This strategy was used to prepare protein–protein conjugates of α-synuclein with either ubiquitin or ISG15, the latter having the same C-terminal peptide sequence as ubiquitin. Protein–protein conjugation via formation of isopeptide bonds offers the opportunity to place the site of conjugation at different parts of the protein sequence, but further work is required to expand the scope of proteins that can participate as the coupling partner beyond ubiquitin-like proteins for a general way to produce protein–protein conjugates.^125^ C-to-C terminal protein–protein conjugation was also achieved by using an engineered asparaginyl ligase and a short bifunctional linker peptide that adds to proteins with a C-terminal sequence tag (NGLH).^126^

Although not strictly an enzymatic method, some protein–protein dimers have been prepared using intein-based methods. Inteins are peptide sequences that can be induced to cleave with concomitant ligation of the flanking peptide sequences (exteins). The pathway proceeds via a thioester intermediate which can be potentially intercepted and used as a component in native chemical (NCL) with another protein bearing a N-terminal cysteine residue.^127^ In series, this process is referred to as express chemical ligation (EPL) and has been used for the preparation the protein–protein conjugate of histone H2B and ubiquitin featuring either the natural isopeptide linkage or a synthetically more tractable disulfide analogue.^128−130^ In addition, Ub–Ub homodimers have been produced for NMR studies.^131^

The formylglycine-generating enzyme (FGE) has been used to convert cysteine residues contained in a CXPXR motif into a formylglycine residue, which bears a reactive aldehyde group in its side chain. Treatment of the aldehyde-containing protein with a bifunctional small molecule bearing (i) an aminooxy group for reaction with the formylglycine group and (ii) either an azide or alkyne group for further modification using click chemistry. Following independent preparation of azide-tagged and alkyne-tagged proteins, heterodimerization was achieved to provide full length human IgG (155 kDa) conjugates with either human growth hormone (26 kDa) or the maltose-binding protein (42 kDa).^100^ Recently, it was found that FGE-mediated protein–protein conjugation could be accelerated by freezing, an effect ostensibly attributed to extreme changes in pH, ionic strength, and liquid water concentration as ice crystals form.^132^

A recent report exhaustively tested different bioorthogonal coupling partners, which were enzymatically installed, for protein–protein conjugation.^21^ The enzyme lipoic acid protein ligase (LAPL) recognizes the 13-residue LAP sequence (Figure 11) and catalyzes the formation of an isopeptide bond between an internal lysine residue in the LAP sequence and a carboxylic acid bearing group in a small molecule probe. The 14 probes used in the study each featured bioorthogonal functional groups and enabled screening of several well-known click reactions leading to protein–protein conjugates. An optimal pairing was found with a tetrazine and strained cyclooctyne (TCO) operating under an IEDDA mechanism with an approximate second-order rate constant of 50 M^–1^ s^–1^ at 37 °C in phosphate buffered saline.

This method was ultimately used to prepare a triple-protein conjugate of trastuzumab; the LAP-tag was added to each of the heavy chain C-termini and the tags were functionalized with GFP. Remarkably, the reaction was quantitative after 4 h using two stoichiometric equivalents of GFP and the product maintained low-nanomolar binding to HER2^+^ cells.^21^

### 5.4 SpyTag/SpyCatcher-Based Methods

**Figure 12:**
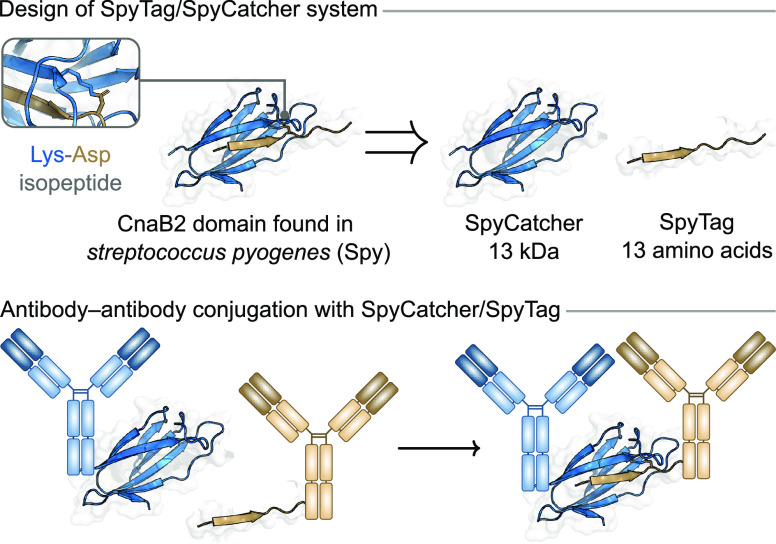
Design of SpyCatcher/SpyTag system and application to preparation of bispecific antibodies.

The SpyTag/SpyCatcher system is not an enzymatic process, but is a popular method for preparing protein–protein conjugates and involves a sequence tag. The system emerged from studies of the second immunoglobulin-like collagen adhesion domain (CnaB2) from the fibronectin binding protein (FbaB) found in *Streptococcus pyogenes* (Spy, Figure 12).^133^ The CnaB2 domain is exceptionally stable, remaining folded after boiling at pH 2, and harbors an isopeptide bond between an aspartic acid and lysine residue. The SpyTag/SpyCatcher conjugation system was designed by splitting the CnaB2 Spy domain into two portions at this isopeptide bond: a tag comprising 13 amino acids (SpyTag) and the remaining protein sequence (SpyCatcher, 13 kDa). Upon mixing of the two fragments, the original CnaB2 domain is rapidly reconstituted. Expression of the SpyTag and SpyCatcher domains into two different proteins results in them forming a stable protein–protein conjugate, linked by the CnaB2 domain.

The SpyTag/SpyCatcher system was used to prepare bispecific antibodies that recognize two different domains of the transmembrane protein roundabout homologue 1 (ROBO1). The protein components that underwent conjugation were each scFv fragments, and the approach ultimately led to a tetravalent bispecific antibody (two scFv fragments per molecule) that displayed midrange picomolar affinity for its target where individual components bind in the mid-to-low nanomolar range.^134,135^

Later work used the SpyCatcher/SpyTag system to build anti-HER3 antibodies from individual building blocks (Fc, Fab, and scFv regions) that each featured an appropriate Spy-based domain for conjugation.^136^ A follow-up study produced a trivalent scFv; the central development that enabled this was synthesis of a peptide comprising three consecutive SpyTag sequences. Treatment of this peptide with an scFv-SpyCatcher fusion protein resulted in the desired anti-HER3 trivalent scFv.^137^

An interesting practical development was recently disclosed that resulted in fewer protein purification steps. Individual proteins bearing respective SpyCatcher and SpyTag domains were expressed in HEK 293F cells; combining the cell culture media at 37 °C for 3 h gave the desired protein–protein conjugate which activated the canonical Wnt signaling pathway.^138^ Further practical improvements were made by developing a protease-knockout variant *E. coli* strain that permits the expression of Spy-tagged Fabs into the periplasm. This enabled Spy-tagged antibody fragments to be used in a modular fashion, with examples such as coupling to Fc regions and enzymes.^139^ Expression of Spy-tagged proteins has also been achieved in silkworms.^140^

Additional applications of protein–protein conjugates generated using the SpyCatcher/SpyTag system include bispecific immune engagers, antibody–enzyme complexes, and bispecific antibodies.^141−143^ A recent DogTag/DogCatcher pair enables this strategy to be applied to internal tag sequences in a loop-friendly manner.^144^ Fast progress has been made, although it was been pointed out that, as a domain of a *Streptococcus* surface protein, SpyCatcher is expected to induce a strong immune response.^139^ This could mean that while the Spy-system is well suited to screening and development, it may need to be replaced by a different conjugation system for therapeutics based on protein–protein conjugates.^139^

## 6 Outlook

Although post-translational approaches for generating protein–protein conjugates have been investigated for several decades, early techniques lacked the site selectivity required to produce well-defined protein–protein conjugates and, therefore, did not endure with the advent of powerful genetic engineering methods for producing fusion proteins. However, the past two decades have witnessed significant progress in site-selective and site-specific conjugation methods, enabling the preparation of protein–protein conjugates with greater control.

In general, utilizing post-translational chemical conjugation methods can overcome many of the challenges associated with fully expression-based, fusion methods. In particular, the ability to achieve any desired topological arrangement of the target conjugates (N-to-N, C-to-C, internal-internal) obviates the requirement for N-to-C terminal ligation imposed by fusion methods. Additionally, proteins can be conjugated at a “late stage” in the overall preparation, leading to the development of some combinatorial techniques.^92,113^

Within the broad field of site-selective bioconjugation, cysteine remains an essential target, and so cysteine-specific reactions are a dominant subfield of protein–protein conjugation. The low abundance and high nucleophilicity of cysteine make it the target of choice for many strategies, from traditional bismaleimide reagents to novel metal-mediated cross-coupling, as well as in some enzymatic and sequence tag-based approaches. Furthermore, even amine-selective strategies, such as those using SPDP or Traut’s reagent, ultimately use the reactivity of thiols to generate the final protein–protein conjugates. This is evidence that cysteine and thiols in general are the favored reactive handles, and it is unlikely that this status quo will change in the near future. However, as more site-specific conjugation strategies targeting other canonical amino acids become available, perhaps a gradual shift away from the reliance on thiol-based methodologies will occur.

Another strength of chemical-based approaches is the diversity of linker motifs that can be generated with variation in chain length, flexibility, hydrophilicity, and their ability to be orthogonally functionalized with small molecules. Although some of these properties can be varied to some extent with peptide-based linkers using recombinant expression, each variation requires starting at the genetic level which can be both challenging and costly. On the other hand, chemical linkers present a wider design space than peptide-based linkers and libraries of pregenerated linkers can be produced and utilized in parallel with the same protein domains (which only require expression once). Studies on “linker-ology” in the field of PROTAC design suggest that exploring linker space in protein–protein conjugates is currently underdeveloped.^145^ Linker design could, for example, play an important role in producing bispecific antibodies by enhancing antigen-binding properties in vivo.

A potential limitation of post-translational linking strategies that requires further exploration is whether certain linkers produce immunogenic properties. Several protein–protein coupling reactions utilize functional groups that are not found in nature, such as DBCO or TCO, and the presence of these moieties as their respective click products could potentially lead to a larger immune response than PEG- or peptide-based linkers. In terms of applications, post-translationally generated protein–protein conjugates have typically been limited to extracellular roles. This is in contrast to genetic fusion proteins, which can be expressed intracellularly in vivo and therefore used to study a wide range of biological functions.

An issue encountered in the course of preparing the present article was the inconsistency as to how conversions and yields were quoted. A consensus needs to be reached in the field on how best to quantify the conjugation efficiency of this class of reactions. We propose that a gold standard would be to quote the isolated yield after an appropriate purification step, such as size exclusion chromatography, of the protein–protein conjugate. While conversion remains a useful descriptor for reaction screening and optimization, it should not be provided as the sole measure of a given conjugation process. Use of a battery of techniques, including circular dichroism and functional assays, should be included in addition to standard analyses via SDS-PAGE or LC–MS. Reporting in line with these principles will allow researchers to select the conjugation technique that is most suitable for their specific requirements. In addition, comparisons between post-translational conjugation strategies (e.g., site-specific cysteine chemistry vs enzymatic conjugation) are typically not performed, and it is unclear whether a particular approach may present a general advantage over another.

Nonetheless, great strides have been taken in developing post-translational strategies for producing protein–protein conjugates. With this in mind, a fruitful period for the field is anticipated, in which many more strategies will be developed and the existing toolbox will be widely utilized to generate protein–protein conjugates that can be used to probe important biological questions.

## References

[ref1] Ciechanover A.; Schwartz A. L. The ubiquitin-proteasome pathway: The complexity and myriad functions of proteins death. Proc. Natl. Acad. Sci. U.S.A. 1998, 95, 2727–2730. 10.1073/pnas.95.6.2727. 9501156 PMC34259

[ref2] Morgan M. T.; Haj-Yahya M.; Ringel A. E.; Bandi P.; Brik A.; Wolberger C. Structural basis for histone H2B deubiquitination by the SAGA DUB module. Science 2016, 351, 725–728. 10.1126/science.aac5681. 26912860 PMC4863942

[ref3] Mali S. M.; Singh S. K.; Eid E.; Brik A. Ubiquitin Signaling: Chemistry Comes to the Rescue. J. Am. Chem. Soc. 2017, 139, 4971–4986. 10.1021/jacs.7b00089. 28328208

[ref4] Sun H.; Brik A. The Journey for the Total Chemical Synthesis of a 53 kDa Protein. Acc. Chem. Res. 2019, 52, 3361–3371. 10.1021/acs.accounts.9b00372. 31536331

[ref5] Yu K.; Liu C.; Kim B.-G.; Lee D.-Y. Synthetic fusion protein design and applications. Biotechnol. Adv. 2015, 33, 155–164. 10.1016/j.biotechadv.2014.11.005. 25450191

[ref6] Bell M. R.; Engleka M. J.; Malik A.; Strickler J. E. To fuse or not to fuse: What is your purpose?. Protein Sci. 2013, 22, 1466–1477. 10.1002/pro.2356. 24038604 PMC3831663

[ref7] Yuste R. Fluorescence microscopy today. Nat. Methods 2005, 2, 902–904. 10.1038/nmeth1205-902. 16299474

[ref8] Iturrate L.; Sánchez-Moreno I.; Oroz-Guinea I.; Pérez-Gil J.; García-Junceda E. Preparation and Characterization of a Bifunctional Aldolase/Kinase Enzyme: A More Efficient Biocatalyst for C-C Bond Formation. Chem. - Eur. J. 2010, 16, 4018–4030. 10.1002/chem.200903096. 20198665

[ref9] Labrijn A. F.; Janmaat M. L.; Reichert J. M.; Parren P. W. H. I. Bispecific antibodies: a mechanistic review of the pipeline. Nat. Rev. Drug Discovery 2019, 18, 585–608. 10.1038/s41573-019-0028-1. 31175342

[ref10] Chen X.; Zaro J. L.; Shen W.-C. Fusion protein linkers: Property, design and functionality. Adv. Drug Delivery Rev. 2013, 65, 1357–1369. 10.1016/j.addr.2012.09.039. PMC3726540 23026637

[ref11] Zhang J.; Yun J.; Shang Z.; Zhang X.; Pan B. Design and optimization of a linker for fusion protein construction. Prog. Nat. Sci. 2009, 19, 1197–1200. 10.1016/j.pnsc.2008.12.007.

[ref12] Witte M. D.; Cragnolini J. J.; Dougan S. K.; Yoder N. C.; Popp M. W.; Ploegh H. L. Preparation of unnatural N-to-N and C-to-C protein fusions. Proc. Natl. Acad. Sci. U.S.A. 2012, 109, 11993–11998. 10.1073/pnas.1205427109. 22778432 PMC3409725

[ref13] Lee B. S.; Lee Y.; Park J.; Jeong B. S.; Jo M.; Jung S. T.; Yoo T. H. Construction of an immunotoxin via site-specific conjugation of anti-Her2 IgG and engineered Pseudomonas exotoxin A. J. Biol. Eng. 2019, 13, 56 10.1186/s13036-019-0188-x. 31285754 PMC6588878

[ref14] Hoyt E. A.; Cal P. M. S. D.; Oliveira B. L.; Bernardes G. J. L. Contemporary approaches to site-selective protein modification. Nat. Rev. Chem. 2019, 3, 147–171. 10.1038/s41570-019-0079-1.

[ref15] Szijj P.; Chudasama V. The renaissance of chemically generated bispecific antibodies. Nat. Rev. Chem. 2021, 5, 78–92. 10.1038/s41570-020-00241-6. 37117612

[ref16] Park J.; Lee S.; Kim Y.; Yoo T. H. Methods to generate site-specific conjugates of antibody and protein. Bioorg. Med. Chem. 2021, 30, 115946 10.1016/j.bmc.2020.115946. 33360577

[ref17] Dimasi N.; Kumar A.; Gao C. Generation of bispecific antibodies using chemical conjugation methods. Drug Discovery Today: Technol. 2021, 40, 13–24. 10.1016/j.ddtec.2021.08.006. 34916015

[ref18] Saito F.; Noda H.; Bode J. W. Critical Evaluation and Rate Constants of Chemoselective Ligation Reactions for Stoichiometric Conjugations in Water. ACS. Chem. Biol. 2015, 10, 1026–1033. 10.1021/cb5006728 25572124

[ref19] Mogilevsky C. S.; Lobba M. J.; Brauer D. D.; Marmelstein A. M.; Maza J. C.; Gleason J. M.; Doudna J. A.; Francis M. B. Synthesis of Multi-Protein Complexes through Charge-Directed Sequential Activation of Tyrosine Residues. J. Am. Chem. Soc. 2021, 143, 13538–13547. 10.1021/jacs.1c03079. 34382787 PMC9274618

[ref20] Lang K.; Chin J. W. Bioorthogonal Reactions for Labeling Proteins. ACS Chem. Bio. 2014, 9, 16–20. 10.1021/cb4009292. 24432752

[ref21] Baalmann M.; Neises L.; Bitsch S.; Schneider H.; Deweid L.; Werther P.; Ilkenhans N.; Wolfring M.; Ziegler M. J.; Wilhelm J.; Kolmar H.; Wombacher R. A Bioorthogonal Click Chemistry Toolbox for Targeted Synthesis of Branched and Well-Defined Protein-Protein Conjugates. Angew. Chem., Int. Ed. 2020, 59, 12885–12893. 10.1002/anie.201915079. PMC7496671 32342666

[ref22] Hvasanov D.; Nam E. V.; Peterson J. R.; Pornsaksit D.; Wiedenmann J.; Marquis C. P.; Thordarson P. One-Pot Synthesis of High Molecular Weight Synthetic Heteroprotein Dimers Driven by Charge Complementarity Electrostatic Interactions. J. Org. Chem. 2014, 79, 9594–9602. 10.1021/jo501713t. 25231623

[ref23] Silvius J. R.; Leventis R. A Novel “Prebinding”Strategy Dramatically Enhances Sortase-Mediated Coupling of Proteins to Liposomes. Bioconj. Chem. 2017, 28, 1271–1282. 10.1021/acs.bioconjchem.7b00087. 28358190

[ref24] Kennedy J. H.; Kricka L. J.; Wilding P. Protein-protein coupling reactions and the applications of protein conjugates. Clin. Chim. Acta 1976, 70, 1–31. 10.1016/0009-8981(76)90002-4. 820493

[ref25] Singer S. J.; Fothergill J. E.; Shainoff J. R. A General Method for the Isolation of Antibodies. J. Am. Chem. Soc. 1960, 82, 565–571. 10.1021/ja01488a018.

[ref26] Moore J. E.; Ward W. H. Cross-linking of Bovine Plasma Albumin and Wool Keratin. J. Am. Chem. Soc. 1956, 78, 2414–2418. 10.1021/ja01592a020.

[ref27] Ozawa H. Bridging Reagent for Protein: I. The Reaction of Diisocyanates with Lysine and Enzyme Proteins. J. Biochem. 1967, 62, 419–423. 10.1093/oxfordjournals.jbchem.a128684. 5587589

[ref28] Habeeb A. F. S. A.; Hiramoto R. Reaction of proteins with glutaraldehyde. Arch. Biochem. Biophys. 1968, 126, 16–26. 10.1016/0003-9861(68)90554-7. 4174905

[ref29] Wold F. Some properties of cross-linked bovine serum albumin. Biochim. Biophys. Acta 1961, 54, 604–606. 10.1016/0006-3002(61)90110-X. 14007909

[ref30] Clyne D. H.; Norris S. H.; Modesto R. R.; Pesce A. J.; Pollak V. E. Antibody Enzyme Conjugates The Preparation of Intermolecular Conjugates of Horseradish Peroxidase and Antibody and their use in Immunohistology of Renal Cortex. J. Histochem. Cytochem. 1973, 21, 233–240. 10.1177/21.3.233. 4121417

[ref31] Wang T.-W.; Kassell B. Preparation of a chemically cross-linked complex of the basic pancreatic trypsin inhibitor with trypsin. Biochemistry 1974, 13, 698–702. 10.1021/bi00701a010. 4855764

[ref32] Davies G. E.; Stark G. R. Use of Dimethyl Suberimidate, a Cross-Linking Reagent, in Studying the Subunit Structure of Oligomeric Proteins. Proc. Natl. Acad. Sci. U.S.A. 1970, 66, 651–656. 10.1073/pnas.66.3.651. 4913206 PMC283100

[ref33] Spicer C. D.; Davis B. G. Selective chemical protein modification. Nat. Commun. 2014, 5, 4740 10.1038/ncomms5740. 25190082

[ref34] Itzkovitz S.; Alon U. The genetic code is nearly optimal for allowing additional information within protein-coding sequences. Genome Res. 2007, 17, 405–412. 10.1101/gr.5987307. 17293451 PMC1832087

[ref35] Glennie M. J.; McBride H. M.; Worth A. T.; Stevenson G. T. Preparation and performance of bispecific F(ab’ gamma)2 antibody containing thioether-linked Fab’ gamma fragments. J. Immunol. 1987, 139, 2367–2375. 2958547

[ref36] Scheer J. M.; Sandoval W.; Elliott J. M.; Shao L.; Luis E.; Lewin-Koh S.-C.; Schaefer G.; Vandlen R. Reorienting the Fab Domains of Trastuzumab Results in Potent HER2 Activators. PLoS One 2012, 7, e51817 10.1371/journal.pone.0051817. 23284778 PMC3527469

[ref37] Ravasco J. M. J. M.; Faustino H.; Trindade A.; Gois P. M. P. Bioconjugation with Maleimides: A Useful Tool for Chemical Biology. Chem. - Eur. J. 2019, 25, 43–59. 10.1002/chem.201803174. 30095185

[ref38] Kane B. J.; Fettis M. M.; Farhadi S. A.; Liu R.; Hudalla G. A. Site-Specific Cross-Linking of Galectin-1 Homodimers via Poly(ethylene glycol) Bismaleimide. Cell. Mol. Bioeng. 2021, 14, 523–534. 10.1007/s12195-021-00681-0. 34777608 PMC8548478

[ref39] Szijj P. A.; Bahou C.; Chudasama V. Minireview: Addressing the retro-Michael instability of maleimide bioconjugates. Drug Discovery Today: Technol. 2018, 30, 27–34. 10.1016/j.ddtec.2018.07.002. 30553517

[ref40] Laserna V.; Istrate A.; Kafuta K.; Hakala T. A.; Knowles T. P. J.; Alcarazo M.; Bernardes G. J. L. Protein Conjugation by Electrophilic Alkynylation Using 5-(Alkynyl)dibenzothiophenium Triflates. Bioconj. Chem. 2021, 32, 1570–1575. 10.1021/acs.bioconjchem.1c00317. 34232618

[ref41] Khalili H.; Godwin A.; Choi J.-w.; Lever R.; Khaw P. T.; Brocchini S. Fab-PEG-Fab as a Potential Antibody Mimetic. Bioconj. Chem. 2013, 24, 1870–1882. 10.1021/bc400246z. 24073593

[ref42] Hull E. A.; Livanos M.; Miranda E.; Smith M. E. B.; Chester K. A.; Baker J. R. Homogeneous Bispecifics by Disulfide Bridging. Bioconj. Chem. 2014, 25, 1395–1401. 10.1021/bc5002467. PMC4458859 25033024

[ref43] Forte N.; Livanos M.; Miranda E.; Morais M.; Yang X.; Rajkumar V. S.; Chester K. A.; Chudasama V.; Baker J. R. Tuning the Hydrolytic Stability of Next Generation Maleimide Cross-Linkers Enables Access to Albumin-Antibody Fragment Conjugates and tri-scFvs. Bioconj. Chem. 2018, 29, 486–492. 10.1021/acs.bioconjchem.7b00795. 29384367

[ref44] Kuan S. L.; Wang T.; Weil T. Site-Selective Disulfide Modification of Proteins: Expanding Diversity beyond the Proteome. Chem. - Eur. J. 2016, 22, 17112–17129. 10.1002/chem.201602298. 27778400 PMC5600100

[ref45] Oliveira B. L.; Guo Z.; Bernardes G. J. L. Inverse electron demand Diels–Alder reactions in chemical biology. Chem. Soc. Rev. 2017, 46, 4895–4950. 10.1039/C7CS00184C. 28660957

[ref46] Natarajan A.; Du W.; Xiong C.-Y.; DeNardo G. L.; DeNardo S. J.; Gervay-Hague J. Construction of di-scFv through a trivalent alkyne–azide 1,3-dipolar cycloaddition. Chem. Commun. 2007, 695–697. 10.1039/B611636A. 17392953

[ref47] Schellinger J. G.; Kudupudi A.; Natarajan A.; Du W.; DeNardo S. J.; Gervay-Hague J. A general chemical synthesis platform for crosslinking multivalent single chain variable fragments. Org. Biomol. Chem. 2012, 10, 1521–1526. 10.1039/C0OB01259A. 22132412 PMC4497512

[ref48] Foot J. S.; Lui F. E.; Kluger R. Hemoglobin bis-tetramers via cooperative azide-alkyne coupling. Chem. Commun. 2009, 7315–7317. 10.1039/b918860f. 20024213

[ref49] Yang Y.; Kluger R. Efficient CuAAC click formation of functional hemoglobin bis-tetramers. Chem. Commun. 2010, 46, 7557–7559. 10.1039/c0cc02023k. 20852763

[ref50] Hatzakis N. S.; Engelkamp H.; Velonia K.; Hofkens J.; Christianen P. C. M.; Svendsen A.; Patkar S. A.; Vind J.; Maan J. C.; Rowan A. E.; Nolte R. J. M. Synthesis and single enzyme activity of a clicked lipase-BSA hetero-dimer. Chem. Commun. 2006, 2012–2014. 10.1039/B516551B. 16767259

[ref51] Patterson J. T.; Isaacson J.; Kerwin L.; Atassi G.; Duggal R.; Bresson D.; Zhu T.; Zhou H.; Fu Y.; Kaufmann G. F. PSMA-targeted bispecific Fab conjugates that engage T cells. Bioorg. Med. Chem. Lett. 2017, 27, 5490–5495. 10.1016/j.bmcl.2017.09.065. 29126850

[ref52] Patterson J. T.; Gros E.; Zhou H.; Atassi G.; Kerwin L.; Carmody L.; Zhu T.; Jones B.; Fu Y.; Kaufmann G. F. Chemically generated IgG2 bispecific antibodies through disulfide bridging. Bioorg. Med. Chem. 2017, 27, 3647–3652. 10.1016/j.bmcl.2017.07.021. 28720505

[ref53] Kujawski M.; Li L.; Bhattacharya S.; Wong P.; Lee W.-H.; Williams L.; Li H.; Chea J.; Poku K.; Bowles N.; Vaidehi N.; Yazaki P.; Shively J. E. Generation of dual specific bivalent BiTEs (dbBIspecific T-cell engaging antibodies) for cellular immunotherapy. BMC Cancer 2019, 19, 882 10.1186/s12885-019-6056-8. 31488104 PMC6727398

[ref54] Istrate A.; Geeson M. B.; Navo C. D.; Sousa B. B.; Marques M. C.; Taylor R. J.; Journeaux T.; Oehler S. R.; Mortensen M. R.; Deery M. J.; Bond A. D.; Corzana F.; Jiménez-Osés G.; Bernardes G. J. L. A Platform for Orthogonal N-Cysteine-Specific Protein Modification Enabled by Cyclopropenone Reagents. J. Am. Chem. Soc. 2022, 144, 10396–10406. 10.1021/jacs.2c02185. 35658467 PMC9490850

[ref55] Lorenzo M. M.; Decker C. G.; Kahveci M. U.; Paluck S. J.; Maynard H. D. Homodimeric Protein–Polymer Conjugates via the Tetrazine–trans–Cyclooctene Ligation. Macromolecules 2016, 49, 30–37. 10.1021/acs.macromol.5b02323. 26949271 PMC4776326

[ref56] White C. J.; Bode J. W. PEGylation and Dimerization of Expressed Proteins under Near Equimolar Conditions with Potassium 2-Pyridyl Acyltrifluoroborates. ACS. Cent. Sci. 2018, 4, 197–206. 10.1021/acscentsci.7b00432. 29532019 PMC5833003

[ref57] Maruani A.; Szijj P. A.; Bahou C.; Nogueira J. C. F.; Caddick S.; Baker J. R.; Chudasama V. A Plug-and-Play Approach for the De Novo Generation of Dually Functionalized Bispecifics. Bioconj. Chem. 2020, 31, 520–529. 10.1021/acs.bioconjchem.0c00002. 32093465

[ref58] Levengood M. R.; Zhang X.; Hunter J. H.; Emmerton K. K.; Miyamoto J. B.; Lewis T. S.; Senter P. D. Orthogonal Cysteine Protection Enables Homogeneous Multi-Drug Antibody–Drug Conjugates. Angew. Chem., Int. Ed. 2017, 129, 751–755. 10.1002/ange.201608292. PMC5299463 27966822

[ref59] Wang T.; Pfisterer A.; Kuan S. L.; Wu Y.; Dumele O.; Lamla M.; Müllen K.; Weil T. Cross-conjugation of DNA, proteins and peptides via a pH switch. Chem. Sci. 2013, 4, 1889–1894. 10.1039/c3sc22015j.

[ref60] Baumann A. L.; Schwagerus S.; Broi K.; Kemnitz-Hassanin K.; Stieger C. E.; Trieloff N.; Schmieder P.; Hackenberger C. P. R. Chemically Induced Vinylphosphonothiolate Electrophiles for Thiol–Thiol Bioconjugations. J. Am. Chem. Soc. 2020, 142, 9544–9552. 10.1021/jacs.0c03426. 32338894

[ref61] Qian F.; Reiter K.; Zhang Y.; Shimp L. S. Jr; Nguyen V.; Aebig J. A.; Rausch K. M.; Zhu D.; Lambert L.; Mullen G. E. D.; Martin L. B.; Long C. A.; Miller L. H.; Narum D. L. Immunogenicity of Self-Associated Aggregates and Chemically Cross-Linked Conjugates of the 42 kDa Plasmodium falciparum Merozoite Surface Protein-1. PLoS One 2012, 7, e36996 10.1371/journal.pone.0036996. 22675476 PMC3366955

[ref62] Gui W.; Davidson G. A.; Zhuang Z. Chemical methods for protein site-specific ubiquitination. RSC Chem. Bio. 2021, 2, 450–467. 10.1039/D0CB00215A. 34381999 PMC8323803

[ref63] Haque M.; Forte N.; Baker J. R. lysine conjugation methods and applications towards antibody–drug conjugates. Chem. Commun. 2021, 57, 10689–10702. 10.1039/D1CC03976H. PMC8516052 34570125

[ref64] Vinogradova E. V.; Zhang C.; Spokoyny A. M.; Pentelute B. L.; Buchwald S. L. Organometallic palladium reagents for cysteine bioconjugation. Nature 2015, 526, 687–691. 10.1038/nature15739. 26511579 PMC4809359

[ref65] Kung K. K.-Y.; Ko H.-M.; Cui J.-F.; Chong H.-C.; Leung Y.-C.; Wong M.-K. Cyclometalated gold(III) complexes for chemoselective cysteine modification via ligand controlled C–S bond-forming reductive elimination. Chem. Commun. 2014, 50, 11899–11902. 10.1039/C4CC04467C. 25154886

[ref66] Messina M. S.; Stauber J. M.; Waddington M. A.; Rheingold A. L.; Maynard H. D.; Spokoyny A. M. Organometallic Gold(III) Reagents for Cysteine Arylation. J. Am. Chem. Soc. 2018, 140, 7065–7069. 10.1021/jacs.8b04115. 29790740 PMC6491213

[ref67] Hanaya K.; Ohata J.; Miller M. K.; Mangubat-Medina A. E.; Swierczynski M. J.; Yang D. C.; Rosenthal R. M.; Popp B. V.; Ball Z. T. Rapid nickel(II)-promoted cysteine S-arylation with arylboronic acids. Chem. Commun. 2019, 55, 2841–2844. 10.1039/C9CC00159J. 30768093

[ref68] Waddington M. A.; Zheng X.; Stauber J. M.; Hakim Moully E.; Montgomery H. R.; Saleh L. M. A.; Král P.; Spokoyny A. M. An Organometallic Strategy for Cysteine Borylation. J. Am. Chem. Soc. 2021, 143, 8661–8668. 10.1021/jacs.1c02206. 34060827 PMC8437308

[ref69] Isenegger P. G.; Davis B. G. Concepts of Catalysis in Site-Selective Protein Modifications. J. Am. Chem. Soc. 2019, 141, 8005–8013. 10.1021/jacs.8b13187. 30974939 PMC6535719

[ref70] Dhanjee H. H.; Saebi A.; Buslov I.; Loftis A. R.; Buchwald S. L.; Pentelute B. L. Protein–Protein Cross-Coupling via Palladium-Protein Oxidative Addition Complexes from Cysteine Residues. J. Am. Chem. Soc. 2020, 142, 9124–9129. 10.1021/jacs.0c03143. 32364380 PMC7586714

[ref71] Dhanjee H. H.; Buslov I.; Windsor I. W.; Raines R. T.; Pentelute B. L.; Buchwald S. L. Palladium–Protein Oxidative Addition Complexes by Amine-Selective Acylation. J. Am. Chem. Soc. 2020, 142, 21237–21242. 10.1021/jacs.0c09180. 33319995 PMC8048385

[ref72] Jbara M.; Pomplun S.; Schissel C. K.; Hawken S. W.; Boija A.; Klein I.; Rodriguez J.; Buchwald S. L.; Pentelute B. L. Engineering Bioactive Dimeric Transcription Factor Analogs via Palladium Rebound Reagents. J. Am. Chem. Soc. 2021, 143, 11788–11798. 10.1021/jacs.1c05666. 34289685

[ref73] Pomplun S.; Jbara M.; Schissel C. K.; Wilson Hawken S.; Boija A.; Li C.; Klein I.; Pentelute B. L. Parallel Automated Flow Synthesis of Covalent Protein Complexes That Can Inhibit MYC-Driven Transcription. ACS. Cent. Sci. 2021, 7, 1408–1418. 10.1021/acscentsci.1c00663. 34471684 PMC8393199

[ref74] Swierczynski M. J.; Ball Z. T. One-Step Protein–Polymer Conjugates from Boronic-Acid-Functionalized Polymers. Bioconj. Chem. 2020, 31, 2494–2498. 10.1021/acs.bioconjchem.0c00516. 33078937

[ref75] Ohata J.; Minus M. B.; Abernathy M. E.; Ball Z. T. Histidine-Directed Arylation/Alkenylation of Backbone N–H Bonds Mediated by Copper(II). J. Am. Chem. Soc. 2016, 138, 7472–7475. 10.1021/jacs.6b03390. 27249339

[ref76] Miller M. K.; Swierczynski M. J.; Ding Y.; Ball Z. T. Boronic Acid Pairs for Sequential Bioconjugation. Org. Lett. 2021, 23, 5334–5338. 10.1021/acs.orglett.1c01624. 34212723

[ref77] Karpovsky B.; Titus J. A.; Stephany D. A.; Segal D. M. Production of target-specific effector cells using hetero-cross-linked aggregates containing anti-target cell and anti-Fc gamma receptor antibodies. J. Exp. Med. 1984, 160, 1686–1701. 10.1084/jem.160.6.1686. 6239899 PMC2187539

[ref78] Segal D. M.; Bast B. J. Production of Bispecific Antibodies. Curr. Protoc. Immunol. 1995, 14, 2.13.1–2.13.16. 10.1002/0471142735.im0213s14 18432765

[ref79] Khaw B.-A.; Tekabe Y.; Johnson L. L. Imaging Experimental Atherosclerotic Lesions in ApoE Knockout Mice: Enhanced Targeting with Z2D3-Anti-DTPA Bispecific Antibody and 99mTc-Labeled Negatively Charged Polymers. J. Nucl. Med. 2006, 47, 868–876. 16644758

[ref80] Vasu C.; Gorla S. R.; Prabhakar B. S.; Holterman M. J. Targeted engagement of CTLA-4 prevents autoimmune thyroiditis. Int. Immunol. 2003, 15, 641–654. 10.1093/intimm/dxg061. 12697664

[ref81] Pillow T. H.; Sadowsky J. D.; Zhang D.; Yu S.-F.; Del Rosario G. D.; Xu K.; He J.; Bhakta S.; Ohri R.; Kozak K. R.; Ha E.; Junutula J. R.; Flygare J. A. Decoupling stability and release in disulfide bonds with antibody-small molecule conjugates. Chem. Sci. 2017, 8, 366–370. 10.1039/C6SC01831A. 28451181 PMC5365059

[ref82] Hakala T. A.; Yates E. V.; Challa P. K.; Toprakcioglu Z.; Nadendla K.; Matak-Vinkovic D.; Dobson C. M.; Martínez R.; Corzana F.; Knowles T. P. J.; Bernardes G. J. L. Accelerating Reaction Rates of Biomolecules by Using Shear Stress in Artificial Capillary Systems. J. Am. Chem. Soc. 2021, 143, 16401–16410. 10.1021/jacs.1c03681. 34606279 PMC8517977

[ref83] Lee R. J.; Fang Q.; Davol P. A.; Gu Y.; Sievers R. E.; Grabert R. C.; Gall J. M.; Tsang E.; Yee M. S.; Fok H.; Huang N. F.; Padbury J. F.; Larrick J. W.; Lum L. G. Antibody Targeting of Stem Cells to Infarcted Myocardium. Stem Cells 2007, 25, 712–717. 10.1634/stemcells.2005-0602. 17138964

[ref84] Reusch U.; Sundaram M.; Davol P. A.; Olson S. D.; Davis J. B.; Demel K.; Nissim J.; Rathore R.; Liu P. Y.; Lum L. G. Anti-CD3 × Anti-Epidermal Growth Factor Receptor (EGFR) Bispecific Antibody Redirects T-Cell Cytolytic Activity to EGFR-Positive Cancers In vitro and in an Animal Model. Clin. Cancer Res. 2006, 12, 183–190. 10.1158/1078-0432.CCR-05-1855. 16397041

[ref85] Chan J. K.; Hamilton C. A.; Cheung M. K.; Karimi M.; Baker J.; Gall J. M.; Schulz S.; Thorne S. H.; Teng N. N.; Contag C. H.; Lum L. G.; Negrin R. S. Enhanced Killing of Primary Ovarian Cancer by Retargeting Autologous Cytokine-Induced Killer Cells with Bispecific Antibodies: A Preclinical Study. Clin. Cancer Res. 2006, 12, 1859–1867. 10.1158/1078-0432.CCR-05-2019. 16551871

[ref86] Gall J. M.; Davol P. A.; Grabert R. C.; Deaver M.; Lum L. G. T cells armed with anti-CD3 × anti-CD20 bispecific antibody enhance killing of CD20+ malignant B cells and bypass complement-mediated rituximab resistance in vitro. Exp. Hematol. 2005, 33, 452–459. 10.1016/j.exphem.2005.01.007. 15781336

[ref87] Ueda A.; Umetsu M.; Nakanishi T.; Hashikami K.; Nakazawa H.; Hattori S.; Asano R.; Kumagai I. Chemically Crosslinked Bispecific Antibodies for Cancer Therapy: Breaking from the Structural Restrictions of the Genetic Fusion Approach. Int. J. Mol. Sci. 2020, 21, 711 10.3390/ijms21030711. PMC7037651 31973200

[ref88] Kölmel D. K.; Kool E. T. Oximes and Hydrazones in Bioconjugation: Mechanism and Catalysis. Chem. Rev. 2017, 117, 10358–10376. 10.1021/acs.chemrev.7b00090. 28640998 PMC5580355

[ref89] Liu J.; Cai L.; Sun W.; Cheng R.; Wang N.; Jin L.; Rozovsky S.; Seiple I. B.; Wang L. Photocaged Quinone Methide Crosslinkers for Light-Controlled Chemical Crosslinking of Protein–Protein and Protein–DNA Complexes. Angew. Chem., Int. Ed. 2019, 58, 18839–18843. 10.1002/anie.201910135. PMC6917869 31644827

[ref90] Saleh A. M.; Wilding K. M.; Calve S.; Bundy B. C.; Kinzer-Ursem T. L. Non-canonical amino acid labeling in proteomics and biotechnology. J. Biol. Eng. 2019, 13, 43 10.1186/s13036-019-0166-3. 31139251 PMC6529998

[ref91] Naowarojna N.; Cheng R.; Lopez J.; Wong C.; Qiao L.; Liu P. Chemical modifications of proteins and their applications in metalloenzyme studies. Synth. Syst. Biotechnol 2021, 6, 32–49. 10.1016/j.synbio.2021.01.001. 33665390 PMC7897936

[ref92] Kim C. H.; Axup J. Y.; Dubrovska A.; Kazane S. A.; Hutchins B. A.; Wold E. D.; Smider V. V.; Schultz P. G. Synthesis of Bispecific Antibodies using Genetically Encoded Unnatural Amino Acids. J. Am. Chem. Soc. 2012, 134, 9918–9921. 10.1021/ja303904e. 22642368 PMC4299457

[ref93] Cao Y.; et al. Multiformat T-Cell-Engaging Bispecific Antibodies Targeting Human Breast Cancers. Angew. Chem., Int. Ed. 2015, 54, 7022–7027. 10.1002/anie.201500799. PMC4492699 25919418

[ref94] Chames P.; Baty D. Bispecific antibodies for cancer therapy: the light at the end of the tunnel?. mAbs 2009, 1, 539–547. 10.4161/mabs.1.6.10015. 20073127 PMC2791310

[ref95] Rutkowska A.; Plass T.; Hoffmann J.-E.; Yushchenko D. A.; Feng S.; Schultz C. T-CrAsH: A Heterologous Chemical Crosslinker. ChemBioChem. 2014, 15, 1765–1768. 10.1002/cbic.201402189. 25045107

[ref96] Hutchins B. M.; Kazane S. A.; Staflin K.; Forsyth J. S.; Felding-Habermann B.; Smider V. V.; Schultz P. G. Selective Formation of Covalent Protein Heterodimers with an Unnatural Amino Acid. Chem. Biol. (Oxford, U. K.) 2011, 18, 299–303. 10.1016/j.chembiol.2011.01.006 PMC3694407 21439474

[ref97] ElSohly A. M.; MacDonald J. I.; Hentzen N. B.; Aanei I. L.; El Muslemany K. M.; Francis M. B. *ortho*-Methoxyphenols as Convenient Oxidative Bioconjugation Reagents with Application to Site-Selective Heterobifunctional Cross-Linkers. J. Am. Chem. Soc. 2017, 139, 3767–3773. 10.1021/jacs.6b12966. 28207247

[ref98] Mazor R.; Onda M.; Pastan I. Immunogenicity of therapeutic recombinant immunotoxins. Immunol. Rev. 2016, 270, 152–164. 10.1111/imr.12390. 26864110 PMC4758696

[ref99] Kim S.; Ko W.; Sung B. H.; Kim S. C.; Lee H. S. Direct protein-protein conjugation by genetically introducing bioorthogonal functional groups into proteins. Bioorg. Med. Chem. 2016, 24, 5816–5822. 10.1016/j.bmc.2016.09.035. 27670101

[ref100] Hudak J. E.; Barfield R. M.; de Hart G. W.; Grob P.; Nogales E.; Bertozzi C. R.; Rabuka D. Synthesis of Heterobifunctional Protein Fusions Using Copper-Free Click Chemistry and the Aldehyde Tag. Angew. Chem., Int. Ed. 2012, 51, 4161–4165. 10.1002/anie.201108130. PMC3379715 22407566

[ref101] Bundy B. C.; Swartz J. R. Site-Specific Incorporation of p-Propargyloxyphenylalanine in a Cell-Free Environment for Direct Protein-Protein Click Conjugation. Bioconj. Chem. 2010, 21, 255–263. 10.1021/bc9002844. 20099875

[ref102] Xu H.; Wang Y.; Lu J.; Zhang B.; Zhang Z.; Si L.; Wu L.; Yao T.; Zhang C.; Xiao S.; Zhang L.; Xia Q.; Zhou D. Re-exploration of the Codon Context Effect on Amber Codon-Guided Incorporation of Noncanonical Amino Acids in Escherichia coli by the Blue–White Screening Assay. ChemBioChem. 2016, 17, 1250–1256. 10.1002/cbic.201600117. 27028123

[ref103] Kato A.; Kuratani M.; Yanagisawa T.; Ohtake K.; Hayashi A.; Amano Y.; Kimura K.; Yokoyama S.; Sakamoto K.; Shiraishi Y. Extensive Survey of Antibody Invariant Positions for Efficient Chemical Conjugation Using Expanded Genetic Codes. Bioconj. Chem. 2017, 28, 2099–2108. 10.1021/acs.bioconjchem.7b00265. 28727448

[ref104] Zhang Y.; Park K.-Y.; Suazo K. F.; Distefano M. D. Recent progress in enzymatic protein labelling techniques and their applications. Chem. Soc. Rev. 2018, 47, 9106–9136. 10.1039/C8CS00537K. 30259933 PMC6289631

[ref105] Lieser R. M.; Yur D.; Sullivan M. O.; Chen W. Site-Specific Bioconjugation Approaches for Enhanced Delivery of Protein Therapeutics and Protein Drug Carriers. Bioconj. Chem. 2020, 31, 2272–2282. 10.1021/acs.bioconjchem.0c00456. 32931255

[ref106] Mao H.; Hart S. A.; Schink A.; Pollok B. A. Sortase-Mediated Protein Ligation: A New Method for Protein Engineering. J. Am. Chem. Soc. 2004, 126, 2670–2671. 10.1021/ja039915e. 14995162

[ref107] Kobashigawa Y.; Kumeta H.; Ogura K.; Inagaki F. Attachment of an NMR-invisible solubility enhancement tag using a sortase-mediated protein ligation method. J. Biomol. NMR 2009, 43, 145 10.1007/s10858-008-9296-5. 19140010

[ref108] Krueger A. T.; Kroll C.; Sanchez E.; Griffith L. G.; Imperiali B. Tailoring Chimeric Ligands for Studying and Biasing ErbB Receptor Family Interactions. Angew. Chem., Int. Ed. 2014, 53, 2662–2666. 10.1002/anie.201307869. PMC4018821 24481645

[ref109] Wagner K.; Kwakkenbos M. J.; Claassen Y. B.; Maijoor K.; Böhne M.; van der Sluijs K. F.; Witte M. D.; van Zoelen D. J.; Cornelissen L. A.; Beaumont T.; Bakker A. Q.; Ploegh H. L.; Spits H. Bispecific antibody generated with sortase and click chemistry has broad antiinfluenza virus activity. Proc. Natl. Acad. Sci. U.S.A. 2014, 111, 16820–16825. 10.1073/pnas.1408605111. 25385586 PMC4250106

[ref110] Bartels L.; et al. A Chemo-enzymatically Linked Bispecific Antibody Retargets T Cells to a Sialylated Epitope on CD43 in Acute Myeloid Leukemia. Cancer Res. 2019, 79, 3372–3382. 10.1158/0008-5472.CAN-18-0189. 31064847

[ref111] Glasgow J. E.; Salit M. L.; Cochran J. R. In Vivo Site-Specific Protein Tagging with Diverse Amines Using an Engineered Sortase Variant. J. Am. Chem. Soc. 2016, 138, 7496–7499. 10.1021/jacs.6b03836. 27280683

[ref112] Moliner-Morro A.; Sheward J.; Karl D.; Perez Vidakovics V.; Murrell L.; McInerney B.; Hanke G. M.; Picomolar L. SARS-CoV-2 Neutralization Using Multi-Arm PEG Nanobody Constructs. Biomolecules 1661, 2020, 10. 10.3390/biom10121661 PMC7764822 33322557

[ref113] Andres F.; Schwill M.; Boersma Y. L.; Plückthun A. High-Throughput Generation of Bispecific Binding Proteins by Sortase A–Mediated Coupling for Direct Functional Screening in Cell Culture. Mol. Cancer Ther. 2020, 19, 1080–1088. 10.1158/1535-7163.MCT-19-0633. 31871271

[ref114] Liu Y.; Tian F.; Shi S.; Deng Y.; Zheng P. Enzymatic Protein–Protein Conjugation through Internal Site Verified at the Single-Molecule Level. J. Phys. Chem. Lett. 2021, 12, 10914–10919. 10.1021/acs.jpclett.1c02767. 34734720

[ref115] van Lith S. A. M.; van Duijnhoven S. M. J.; Navis A. C.; Leenders W. P. J.; Dolk E.; Wennink J. W. H.; van Nostrum C. F.; van Hest J. C. M. Legomedicine—A Versatile Chemo-Enzymatic Approach for the Preparation of Targeted Dual-Labeled Llama Antibody-Nanoparticle Conjugates. Bioconj. Chem. 2017, 28, 539–548. 10.1021/acs.bioconjchem.6b00638. PMC5330650 28045502

[ref116] Bruins J. J.; van de Wouw C.; Wagner K.; Bartels L.; Albada B.; van Delft F. L. Highly Efficient Mono-Functionalization of Knob-in-Hole Antibodies with Strain-Promoted Click Chemistry. ACS Omega 2019, 4, 11801–11807. 10.1021/acsomega.9b01727. 31460288 PMC6682001

[ref117] Minamihata K.; Goto M.; Kamiya N. Protein Heteroconjugation by the Peroxidase-Catalyzed Tyrosine Coupling Reaction. Bioconj. Chem. 2011, 22, 2332–2338. 10.1021/bc200420v. 21999311

[ref118] Permana D.; Minamihata K.; Goto M.; Kamiya N. Laccase-catalyzed bioconjugation of tyrosine-tagged functional proteins. J. Biosci. Bioeng. 2018, 126, 559–566. 10.1016/j.jbiosc.2018.05.013. 29903678

[ref119] Minamihata K.; Goto M.; Kamiya N. Site-specific conjugation of an antibody-binding protein catalyzed by horseradish peroxidase creates a multivalent protein conjugate with high affinity to IgG. Biotechnol. J. 2015, 10, 222–226. 10.1002/biot.201400512. 25381877

[ref120] Minamihata K.; Tanaka Y.; Santoso P.; Goto M.; Kozome D.; Taira T.; Kamiya N. Orthogonal Enzymatic Conjugation Reactions Create Chitin Binding Domain Grafted Chitinase Polymers with Enhanced Antifungal Activity. Bioconj. Chem. 2021, 32, 1688–1698. 10.1021/acs.bioconjchem.1c00235. 34251809

[ref121] Minamihata K.; Yamaguchi S.; Nakajima K.; Nagamune T. Tyrosine Coupling Creates a Hyperbranched Multivalent Protein Polymer Using Horseradish Peroxidase via Bipolar Conjugation Points. Bioconj. Chem. 2016, 27, 1348–1359. 10.1021/acs.bioconjchem.6b00138. 27093089

[ref122] Lobba M. J.; Fellmann C.; Marmelstein A. M.; Maza J. C.; Kissman E. N.; Robinson S. A.; Staahl B. T.; Urnes C.; Lew R. J.; Mogilevsky C. S.; Doudna J. A.; Francis M. B. Site-Specific Bioconjugation through Enzyme-Catalyzed Tyrosine–Cysteine Bond Formation. ACS. Cent. Sci. 2020, 6, 1564–1571. 10.1021/acscentsci.0c00940. 32999931 PMC7517114

[ref123] Geeson M. B.; Bernardes G. J. L. Protein–Protein Conjugates: Tyrosine Delivers. ACS. Cent. Sci. 2020, 6, 1473–1475. 10.1021/acscentsci.0c01008. 32999919 PMC7517117

[ref124] Stengl A.; Gerlach M.; Kasper M.-A.; Hackenberger C. P. R.; Leonhardt H.; Schumacher D.; Helma J. TuPPL: Tub-tag mediated C-terminal protein–protein-ligation using complementary click-chemistry handles. Org. Biomol. Chem. 2019, 17, 4964–4969. 10.1039/C9OB00508K. 30932115

[ref125] Hofmann R.; Akimoto G.; Wucherpfennig T. G.; Zeymer C.; Bode J. W. Lysine acylation using conjugating enzymes for site-specific modification and ubiquitination of recombinant proteins. Nat. Chem. 2020, 12, 1008–1015. 10.1038/s41557-020-0528-y. 32929246

[ref126] Rehm F. B. H.; Tyler T. J.; de Veer S. J.; Craik D. J.; Durek T. Enzymatic C-to-C Protein Ligation. Angew. Chem., Int. Ed. 2022, 61, e202116672 10.1002/anie.202116672. PMC9303898 35018698

[ref127] Kulkarni S. S.; Sayers J.; Premdjee B.; Payne R. J. Rapid and efficient protein synthesis through expansion of the native chemical ligation concept. Nat. Rev. Chem. 2018, 2, 0122 10.1038/s41570-018-0122.

[ref128] Muir T. W.; Sondhi D.; Cole P. A. Expressed protein ligation: A general method for protein engineering. Proc. Natl. Acad. Sci. U.S.A. 1998, 95, 6705–6710. 10.1073/pnas.95.12.6705. 9618476 PMC22605

[ref129] McGinty R. K.; Kim J.; Chatterjee C.; Roeder R. G.; Muir T. W. Chemically ubiquitylated histone H2B stimulates hDot1L-mediated intranucleosomal methylation. Nature 2008, 453, 812–816. 10.1038/nature06906. 18449190 PMC3774535

[ref130] Chatterjee C.; McGinty R. K.; Fierz B.; Muir T. W. Disulfide-directed histone ubiquitylation reveals plasticity in hDot1L activation. Nat. Chem. Bio. 2010, 6, 267–269. 10.1038/nchembio.315. 20208522

[ref131] Castañeda C. A.; Spasser L.; Bavikar S. N.; Brik A.; Fushman D. Segmental Isotopic Labeling of Ubiquitin Chains To Unravel Monomer-Specific Molecular Behavior. Angew. Chem., Int. Ed. 2011, 50, 11210–11214. 10.1002/anie.201104649. PMC3427647 21957015

[ref132] Zang B.; Ren J.; Li D.; Huang C.; Ma H.; Peng Q.; Ji F.; Han L.; Jia L. Freezing-assisted synthesis of covalent C–C linked bivalent and bispecific nanobodies. Org. Biomol. Chem. 2019, 17, 257–263. 10.1039/C8OB02323A. 30357229

[ref133] Zakeri B.; Fierer J. O.; Celik E.; Chittock E. C.; Schwarz-Linek U.; Moy V. T.; Howarth M. Peptide tag forming a rapid covalent bond to a protein, through engineering a bacterial adhesin. Proc. Natl. Acad. Sci. U.S.A. 2012, 109, E690–E697. 10.1073/pnas.1115485109. 22366317 PMC3311370

[ref134] Yumura K.; Akiba H.; Nagatoishi S.; Kusano-Arai O.; Iwanari H.; Hamakubo T.; Tsumoto K. Use of SpyTag/SpyCatcher to construct bispecific antibodies that target two epitopes of a single antigen. J. Biochem. 2017, 162, 203–210. 10.1093/jb/mvx023. 28637250

[ref135] Akiba H.; Takayanagi K.; Kusano-Arai O.; Iwanari H.; Hamakubo T.; Tsumoto K. Generation of biparatopic antibody through two-step targeting of fragment antibodies on antigen using SpyTag and SpyCatcher. Biotechnol. Rep. 2020, 25, e00418 10.1016/j.btre.2020.e00418. PMC6976922 31993343

[ref136] Alam M. K.; Gonzalez C.; Hill W.; El-Sayed A.; Fonge H.; Barreto K.; Geyer C. R. Synthetic Modular Antibody Construction by Using the SpyTag/SpyCatcher Protein-Ligase System. ChemBioChem. 2017, 18, 2217–2221. 10.1002/cbic.201700411. 28891272

[ref137] Alam M. K.; Brabant M.; Viswas R. S.; Barreto K.; Fonge H.; Ronald Geyer C. A novel synthetic trivalent single chain variable fragment (tri-scFv) construction platform based on the SpyTag/SpyCatcher protein ligase system. BMC Biotechnol. 2018, 18, 55 10.1186/s12896-018-0466-6. 30200951 PMC6131909

[ref138] Mukherjee A.; Stathos M. E.; Varner C.; Arsiwala A.; Frey S.; Hu Y.; Smalley D. M.; Schaffer D. V.; Kane R. S. One-pot synthesis of heterodimeric agonists that activate the canonical Wnt signaling pathway. Chem. Commun. 2020, 56, 3685–3688. 10.1039/D0CC00920B. PMC7179975 32119023

[ref139] Hentrich C.; Kellmann S.-J.; Putyrski M.; Cavada M.; Hanuschka H.; Knappik A.; Ylera F. Periplasmic expression of SpyTagged antibody fragments enables rapid modular antibody assembly. Cell Chem. Bio. 2021, 28, 813–824.e6. 10.1016/j.chembiol.2021.01.011. 33529581

[ref140] Xu J.; Kato T.; Park E. Y. Development of SpyTag/SpyCatcher-Bacmid Expression Vector System (SpyBEVS) for Protein Bioconjugations Inside of Silkworms. Int. J. Mol. Sci. 2019, 20, 4228 10.3390/ijms20174228. PMC6747175 31470538

[ref141] Nordmaj M. A.; et al. Development of a bispecific immune engager using a recombinant malaria protein. Cell Death Dis. 2021, 12, 1–11. 10.1038/s41419-021-03611-0. 33824272 PMC8024270

[ref142] Kimura H.; Asano R.; Tsukamoto N.; Tsugawa W.; Sode K. Convenient and Universal Fabrication Method for Antibody-Enzyme Complexes as Sensing Elements Using the SpyCatcher/SpyTag System. Anal. Chem. 2018, 90, 14500–14506. 10.1021/acs.analchem.8b04344. 30427170

[ref143] Mei L.; Zappala F.; Tsourkas A. Rapid Production of Bispecific Antibodies from Off-the-Shelf IgGs with High Yield and Purity.. Bioconj. Chem. 2022, 33, 134–141. 10.1021/acs.bioconjchem.1c00476. PMC9104846 34894663

[ref144] Keeble A. H.; Yadav V. K.; Ferla M. P.; Bauer C. C.; Chuntharpursat-Bon E.; Huang J.; Bon R. S.; Howarth M. DogCatcher allows loop-friendly protein-protein ligation. Cell Chem. Biol. 2022, 29, 339–350.e10. 10.1016/j.chembiol.2021.07.005. 34324879 PMC8878318

[ref145] Békés M.; Langley D. R.; Crews C. M. PROTAC targeted protein degraders: the past is prologue. Nat. Rev. Drug Discovery 2022, 21, 181–200. 10.1038/s41573-021-00371-6. 35042991 PMC8765495

